# Protein Kinase R Restricts the Intracellular Survival of *Mycobacterium tuberculosis* by Promoting Selective Autophagy

**DOI:** 10.3389/fmicb.2020.613963

**Published:** 2021-01-22

**Authors:** Robin Smyth, Stefania Berton, Nusrah Rajabalee, Therese Chan, Jim Sun

**Affiliations:** ^1^ Department of Biochemistry, Microbiology and Immunology, University of Ottawa, Ottawa, ON, Canada; ^2^ Centre for Infection, Immunity and Inflammation, University of Ottawa, Ottawa, ON, Canada

**Keywords:** Mycobacterium tuberculosis, macrophage signaling, autophagy, host-directed therapy, Protein Kinase R

## Abstract

Tuberculosis (TB) is a deadly infectious lung disease caused by the pathogenic bacterium *Mycobacterium tuberculosis* (Mtb). The identification of macrophage signaling proteins exploited by Mtb during infection will enable the development of alternative host-directed therapies (HDT) for TB. HDT strategies will boost host immunity to restrict the intracellular replication of Mtb and therefore hold promise to overcome antimicrobial resistance, a growing crisis in TB therapy. Protein Kinase R (PKR) is a key host sensor that functions in the cellular antiviral response. However, its role in defense against intracellular bacterial pathogens is not clearly defined. Herein, we demonstrate that expression and activation of PKR is upregulated in macrophages infected with Mtb. Immunological profiling of human THP-1 macrophages that overexpress PKR (THP-PKR) showed increased production of IP-10 and reduced production of IL-6, two cytokines that are reported to activate and inhibit IFNγ-dependent autophagy, respectively. Indeed, sustained expression and activation of PKR reduced the intracellular survival of Mtb, an effect that could be enhanced by IFNγ treatment. We further demonstrate that the enhanced anti-mycobacterial activity of THP-PKR macrophages is mediated by a mechanism dependent on selective autophagy, as indicated by increased levels of LC3B-II that colocalize with intracellular Mtb. Consistent with this mechanism, inhibition of autophagolysosome maturation with bafilomycin A1 abrogated the ability of THP-PKR macrophages to limit replication of Mtb, whereas pharmacological activation of autophagy enhanced the anti-mycobacterial effect of PKR overexpression. As such, PKR represents a novel and attractive host target for development of HDT for TB, and our data suggest value in the design of more specific and potent activators of PKR.

## Introduction

*Mycobacterium tuberculosis* (Mtb) is responsible for 1.5 million deaths each year and remains the leading cause of infectious disease-related deaths worldwide (WHO, 2020). Due to the emergence of antibiotic-resistant tuberculosis (TB), the development of alternative anti-TB therapeutics is urgently needed. Host-directed therapy (HDT) is a promising treatment strategy, since it aims to boost the host immune response to Mtb rather than targeting the bacterium itself, thereby possessing the potential to circumvent the development of antibiotic resistance.

It has been observed that nearly half of individuals in close contact with highly active TB patients do not produce antibodies against Mtb (Morrison et al., 2008). This suggests that a strong innate immune response can successfully clear Mtb in certain individuals. Since alveolar macrophages are the first line of defense against inhaled bacteria, the persistence of Mtb is largely determined by the bactericidal capacity of macrophages (Behar et al., 2010). As such, the ability of certain individuals to achieve early clearance of Mtb may be due to an enhanced antibacterial response by their macrophages. Targeting host proteins to boost the antibacterial activity of macrophages could therefore be a promising strategy for HDT.

Double stranded RNA-activated protein kinase R (PKR) is one such host protein that has been suggested as a prime candidate for HDT against TB infection (Wu et al., 2012; Tobin, 2015). PKR is a serine/threonine kinase encoded by the human *EIF2AK2* gene and is well characterized for its role in defense against viral infections (García et al., 2007). Transcription of PKR is stimulated by type I interferons (IFN), and the canonical activator of PKR is double-stranded RNA (dsRNA; Zhang et al., 2001). PKR binds viral dsRNA, triggering dimerization and subsequent autophosphorylation events that lead to the activation of the kinase (Zhang et al., 2001). Activated PKR phosphorylates its substrate, eukaryotic translation initiation factor EIF2α, which inhibits mRNA protein translation to prevent viral replication (Dey et al., 2005). PKR is also reported to induce stress-activated apoptosis during viral infection or serum starvation (García et al., 2006), and it has been shown to regulate pyroptosis and necroptosis (Lu et al., 2012; Thapa et al., 2013). The role of PKR in controlling cell death pathways suggests that it may be a promising target for TB HDT, since the specific mode of cell death that occurs in Mtb-infected macrophages largely influences the progression of infection (Behar et al., 2010).

PKR has also been demonstrated to play a role in autophagy. Autophagy was traditionally described as a homeostatic process that generates nutrients by degrading cytoplasmic constituents. However, there is rapidly accumulating evidence that autophagy also plays an important role in immunity. In addition to organelles and proteins, it is now known that the autophagy process can degrade intracellular pathogens (Gutierrez et al., 2004; Nakagawa et al., 2004). Importantly, PKR is required for autophagic degradation of Herpes Simplex Virus-1 (Tallóczy et al., 2006) and activates autophagy in macrophages during parasitic infection (Ogolla et al., 2013). Autophagy is an important defense mechanism in macrophages infected with Mtb, since it can target and degrade cytosolic Mtb after it escapes the phagosome (Watson et al., 2012). Indeed, autophagy induction in Mtb-infected macrophages allows for progressive elimination of the bacteria (Singh et al., 2006), decreased Mtb burden (Gutierrez et al., 2004), and improved control of inflammation (Zhang et al., 2017). Interestingly, current anti-tuberculosis drugs have been shown to activate autophagy. The first-line antibiotics pyrazinamide and isoniazid activate autophagy in Mtb-infected macrophages, and inhibition of autophagy reduces the effectiveness of these drugs (Kim et al., 2012). Due to the antibacterial role of autophagy in macrophages, therapeutic activation of autophagy is a promising HDT strategy against TB (Tobin, 2015; Paik et al., 2019). However, a potential role for PKR in regulating autophagy during bacterial infections has not been studied.

Given that PKR regulates several key macrophage defense mechanisms that are critical for Mtb clearance, PKR could be a promising target for TB HDT. However, knowledge of the function of PKR in macrophages during bacterial infection is surprisingly limited. The impact of PKR on the antibacterial response of macrophages during Mtb infection must therefore be investigated to assess its suitability as a candidate for HDT against TB infection. Herein, we demonstrate that PKR expression and activation is induced during Mtb infection in THP-1 macrophages and primary human macrophages. Through genetic overexpression of PKR, we determined that PKR enhances the production of antibacterial cytokines and limits the intracellular survival of Mtb in macrophages. Our data reveal that PKR enhances the anti-mycobacterial response of macrophages through a mechanism dependent on activation of selective autophagy, and not by manipulation of cell death pathways. As such, a search for pharmacological activators of PKR as a novel TB therapeutic would be desirable.

## Materials and Methods

### Cell Culture and Reagents

THP-1 monocytes (ATCC TIB-202) and primary human monocytes were maintained in RPMI 1640 medium (Gibco, Gaithersburg, MD). HEK GP-293 cells (Clontech, Mountain View, CA) and HEK293T cells (ATCC CRL-3216) were maintained in DMEM medium (Gibco). RPMI 1640 and DMEM medium were supplemented with 2 mM L-glutamine, Penicillin-Streptomycin (100 I.U./ml penicillin, 100 μg/ml streptomycin), 10 mM HEPES, and 10% heat-inactivated fetal bovine serum purchased from Gibco. Cells were maintained at 37°C in a humidified atmosphere of 5% CO_2_. Human peripheral blood mononuclear cells were collected according to approved ethics protocols (Protocol# 2005388-01H) and isolated from buffy coats by the Ficoll-Paque density centrifugation method. Positive selection of monocytes was performed using anti-CD14 coated magnetic particles from StemCell Technologies (Vancouver, BC) according to manufacturer’s protocol. Monocytes were then differentiated with 5 ng/ml GM-CSF (Gibco) for 6 days to obtain human monocyte-derived macrophages (MDMs). THP-1 monocytes were differentiated with 100 ng/ml phorbol ester 13-phorbol-12-myristate acetate (PMA, Alfa Aesar, Haverhill, MA) for 72 h. Puromycin and recombinant human IFNγ were purchased from Gibco. Bafilomycin A1 was purchased from Santa Cruz Biotechnology (Dallas, TX). Rapamycin was purchased from Alfa Aesar.

### Bacteria and Plasmids

The *Mycobacterium tuberculosis* H37Rv-derived auxotroph strain mc^2^6206 was grown in Middlebrook 7H9 medium (BD Biosciences, Franklin Lakes, NJ) supplemented with 0.2% glycerol (Fisher Chemical, Waltham, MA), 0.05% Tween-80 (Acros Organics, Fair Lawn, NJ), 10% OADC (BD Biosciences), 24 μg/ml D-pantothenic acid (Alfa Aesar), and 50 μg/ml L-leucine (Alfa Aesar). *M. tuberculosis* mc^2^6206 expressing green fluorescent protein (GFP; Mtb-GFP) was generated previously (Sun et al., 2016). *M. tuberculosis* mc^2^6206 expressing luciferase (Mtb-luciferase) was generated by transforming the pSMT3 plasmid encoding for firefly luciferase gene (generous gift from Dr. Zakaria Hmama). GFP-expressing and luciferase-expressing Mtb were maintained in antibiotic selection with 50 μg/ml Hygromycin B (Calbiochem, San Diego, CA). Liquid Mtb cultures were maintained at 37°C with slow shaking (50 rpm). *Salmonella enterica* serovar Typhimurium strain SL1344 and *Listeria monocytogenes* strain 10403s were grown in Luria-Bertani broth (Fisher BioReagents, Waltham, MA) at 37°C. *Escherichia coli* strain NEB Stable (New England Biolabs, Ipswich, MA) was used for plasmid propagation and was grown in Luria-Bertani broth at 37°C. Plasmid pMSCV-PKR was constructed by inserting the PCR amplified *EIF2AK2* gene (Entrez Gene ID 5610, variant 1) from THP-1 cell-derived cDNA using the oligonucleotide pair 5'-gctaCTCGAGatggctggtgatctttcagcaggtttc and 5'-gctaGTTAACctaacatgtgtgtcgttcatttttctctg flanked by the XhoI and HpaI restriction sites (capitalized), respectively, into the multiple cloning site of pMSCV-puro (Clontech) for retroviral expression. The CRISPR/Cas9 knock-out plasmid targeting human PKR was generated by inserting the annealed single guide RNA (sgRNA) oligonucleotide pair 5'-agctgttgagatacttaata and 5'-tattaagtatctcaacagct into a modified LentiCRISPR v2 vector (pSL50) linearized by the restriction enzyme BsmBI. The sgRNA was designed to target exon 2 of the human *EIF2AK2* gene and off-target binding was minimized using publicly available online design tools. To optimize the CRISPR/Cas9 system, we performed modifications to the LentiCRISPR v2 vector by creating an A-U base pair flip in the sgRNA stem-loop and by extending the Cas9-binding hairpin structure, as demonstrated by Chen et al. (2013), which resulted in the plasmid pSL50. LentiCRISPR v2 was a gift from Feng Zhang (Addgene plasmid #52961; RRID:Addgene_52961; Sanjana et al., 2014).^1^ The generated plasmids were verified by Sanger sequencing.

### Generation of THP-PKR, THP-Ø, and THP-∆PKR Cells

HEK GP-293 or HEK 293T cells were seeded at 60% confluency to produce viral supernatant. Retroviral plasmids (empty pMSCV-puro and pMSCV-PKR) were co-transfected with the pVSVG envelope plasmid into HEK GP-293 cells, whereas the pSL50 containing the sgRNA targeting PKR was co-transfected with the pVSVG envelope plasmid and the psPAX2 packaging plasmid (Gift from Didier Trono, Addgene plasmid # 12260; RRID:Addgene_12260) into HEK 293T cells.^2^ FuGENE (Promega, Madison, WI) was used as the transfection reagent at a ratio of 4:1 (FuGENE:DNA). Culture supernatants were harvested after 48 h, aliquoted, and stored at −80°C. The supernatants containing retroviral or lentiviral particles were supplemented with 10 μg/ml DEAE-Dextran (Sigma-Aldrich, St. Louis, MO) and used to transduce THP-1 cells. Cells were selected using 1 μg/ml puromycin and analyzed by western blot to verify overexpression or deletion of PKR protein.

### Bacterial Infection

*Mycobacterium tuberculosis* mc^2^6206 growing in log-phase was quantified by optical density measurement at 600 nm using the conversion of OD 1 = 3 × 10^8^ Mtb bacteria per ml. The amount of bacteria required for various multiplicity of infections (MOIs) was washed and resuspended in RPMI 1640 cell culture media without antibiotics. Bacteria were added to the differentiated THP-1 macrophages or primary human MDMs and the cells were incubated at 37°C for 4 h. Three phosphate buffered saline (PBS) washes were then performed to remove extracellular, non-phagocytosed bacteria and infection was continued at 37°C for the desired time. For infections with *S. Typhimurium* and *L. monocytogenes*, frozen stocks with pre-determined CFU/ml were thawed and the amount of bacteria required for various MOIs was resuspended in RPMI 1640 media without antibiotics. The bacteria were added to THP-1 macrophages and incubated at 37°C for 30 min. After 30 min, the extracellular bacteria were removed by performing three PBS washes and the cells were cultured in RPMI medium containing 50 μg/ml gentamicin for 1.5 h. After 1.5 h incubation, cells were washed three times with PBS and cultured in RPMI medium containing 10 μg/ml gentamicin for the remainder of the experiment.

### Macrophage Viability Assay

Serial dilutions of bafilomycin A1 or rapamycin were added to THP-1 macrophages and maintained in the medium for the duration of the experiments. The compounds were replenished every second day. At the indicated time-points, 30 μl of 0.02% resazurin (Sigma-Aldrich) diluted in PBS was added to the wells. After a 4 h incubation at 37°C, fluorescence was measured using the Synergy™ H1 Hybrid Multi-Mode Reader (BioTek, Winooski, VT) with an excitation wavelength of 560 nm and an emission wavelength of 590 nm. Cell cytotoxicity was assessed by comparing the fluorescence of treated cells to untreated cells.

### Intracellular Mtb Survival Assay

Cell culture supernatant was removed from the infected wells and the macrophages were lysed in Glo Lysis Buffer (Promega) at the indicated days post-infection to measure the amount of viable Mtb in each well. Luciferase activity, proportional to viable bacteria, was determined using the BrightGlo Luciferase Assay System (Promega) according to the manufacturer’s protocol. Resultant luminescence was measured with the Synergy™ H1 Hybrid Multi-Mode Microplate Reader (BioTek) using 96-well solid white plates (Corning, Corning, NY) and an integration time of 1 s per well. The linear relationship between luminescence and viable bacteria [evaluated by colony forming units (CFU)] was experimentally confirmed. Briefly, 10-fold serial dilutions of pre-determined Mtb stocks were made in triplicate. The Mtb dilutions were then in part assayed to measure the luciferase activity and in part inoculated on Middlebrook 7H10 (7H10) medium for the detection of CFU by plating. Luciferase activity was determined using the BrightGlo Luciferase Assay System according to the manufacturer’s protocol. Resultant luminescence was measured with the Synergy H1 Hybrid Multi-Mode Microplate Reader using 96-well solid white plates and an integration time of 1 s per well. For CFU detection by plating, bacteria were spread on 7H10 agar plates (with Hygromycin B) and incubated at 37°C for 3 weeks before colony counting was performed.

### Real-Time Cell Analysis Assay

Macrophage adhesion was measured in specialized 96-well plates (E-plate 96) with the xCELLigence Real-time Cell Analyzer (RTCA) SP apparatus (ACEA Biosciences, San Diego, CA). Data was quantified by measuring impedance changes between the sensing electrodes located in the well-bottom, which changes as a function of the adhesion of cells to the surface of the plate. The Cell Index (CI) is a dimensionless value that is representative of these impedance changes. Using this system, macrophage adhesion and therefore viability was monitored in real-time. Plates were removed after ~72 h of macrophage differentiation for the addition of Mtb for infection. After addition of Mtb, plates were placed back in the RTCA apparatus for kinetic monitoring. The CI at every time point represents the mean of three biological replicates.

### Apoptosis Assay

Supernatant was collected from the sample wells to preserve detached cells. The wells were washed twice with PBS and TrypLE Express Enzyme (Gibco) was added to the wells. The plate was incubated at 37°C for 5 min to allow for detachment of adherent THP-1 macrophages. The floating and harvested cells were combined and washed twice with PBS. Cells were stained with Annexin V conjugated to FITC (eBioscience, San Diego, CA) according to manufacturer’s protocol and flow cytometric analysis was used to measure apoptotic cells.

### Multiplex Cytokine and Chemokine Analysis

Cytokine and chemokine expression was measured in culture supernatants harvested from Mtb-infected THP-1 macrophages at 24 h after infection. The LEGENDplex™ Human Essential Immune Response bead-based multiplex assay (BioLegend, San Diego, CA) was used according to the manufacturer’s protocol to measure expression of the following cytokines: IL-10 (0.77 + 1.18), TGF-β (3.10 + 2.92), IL-1β (0.65 + 0.47), TNFα (0.88 + 0.27), IFNγ (0.76 + 0.53), IP-10 (1.28 + 0.48), IL-6 (0.97 + 1.46), IL-8 (1.90 + 0.65), IL-2 (1.81 + 0.93), IL-4 (0.97 + 0.83), IL-17A (2.02 + 0.04), and MCP-1 (1.45 + 0.27). The minimum detectable concentration (MDC) in pg/ml for each cytokine is reported in brackets as MDC + 2 STDEV. Data analysis was performed using LEGENDplex™ Data Analysis Software Version 7.1 (BioLegend).

### Flow Cytometry

Flow cytometric analysis was performed using the CytoFLEX (Beckman Coulter, Indianapolis, IN). Data analysis was performed using CytExpert software (Beckman Coulter) or FlowJo V10 software (BD Life Sciences, Ashland, OR). Flow cytometry was performed to measure cell density and viability using scattering properties. Flow cytometry was also performed to measure cytokine expression, apoptosis, and phagocytosis levels in macrophages.

### Quantitative Real-Time PCR

Total RNA was isolated from THP-1 or primary macrophages using the Aurum Total RNA Mini Kit from Bio-Rad (Hercules, CA). 500 ng of total RNA was used in the cDNA synthesis reaction using the iScript Reverse Transcription Supermix (Bio-Rad). About 4 μl of synthesized cDNA (out of 10 μl reaction) was used to analyze gene expression of *EIF2AK2* or the reference genes *ACTB* and *GAPDH* by real-time PCR on a CFX96 Touch Real-Time PCR Detection System (Bio-Rad) using custom primers that were designed according to MIQE guidelines (Bustin et al., 2009) in combination with the SsoAdvanced Universal SYBR Green Supermix (Bio-Rad). Thermocycling parameters were 95°C for 3 min, followed by 40 cycles of 95°C for 10 s, 60°C for 20 s, and 72°C for 20 s. Gene expression was determined using the ΔΔCq method (Livak and Schmittgen, 2001). ΔCq values were obtained by normalizing the Cq values of *EIF2AK2* with the geometric mean of two reference genes (*ACTB* and *GAPDH*). Relative fold expression was estimated as 2^−ΔΔCq when normalizing to uninfected (day 0) macrophages as 1.0. The following primer pairs were used: *EIF2AK2* forward primer 5'GAAGTGGACCTCTACGCTTTGG and reverse primer 5'TGATGCCATCCCGTAGGTCTGT, *ACTB* forward primer 5'ATTGCCGACAGGATGCAGAA and reverse primer 5'GCTGATCCACATCTGCTGGAA, and *GAPDH* forward primer 5'CAACAGCGACACCCACTCCT and reverse primer 5'CACCCTGTTGCTGTAGCCAAA.

### Western Blot

Macrophages were washed once with PBS and lysed in the wells using RIPA buffer (Sigma-Aldrich) according to manufacturer’s instructions. Protein concentration of the lysates was determined using the RC DC™ Protein Assay Kit (Bio-Rad) according to the manufacturer’s protocol. About 20 μg of protein per sample was separated by SDS-PAGE using handcast 10% polyacrylamide gels (SureCast Gel Handcast System, Invitrogen, Carlsbad, CA) or precast 4–15% polyacrylamide gels (Mini-PROTEAN® TGX Gels, Bio-Rad) and subsequently transferred to a polyvinylidene diluoride (PVDF) membrane using the Mini Trans-Blot Transfer Cell system (Bio-Rad). Primary monoclonal rabbit antibodies to PKR (D7F7), LC3B (D11), and p-S403 p62 (D8D6T) were purchased from Cell Signaling Technology (Danvers, MA). Primary monoclonal rabbit antibody to p-T446 PKR (E120) was purchased from ABCAM (Cambridge, MA). Primary monoclonal mouse antibodies to GAPDH (GA1R) and beta tubulin (BT7R) were purchased from Invitrogen, and primary monoclonal mouse antibody to p62 (D-3) was purchased from Santa Cruz Biotechnology. A horseradish peroxidase-conjugated goat anti-rabbit or anti-mouse polyclonal antibody (Bio-Rad) was used as the secondary antibody. The blot was developed using the Clarity Western Enhanced Chemiluminescence Substrate (Bio-Rad) and detected in an ImageQuant LAS 4000 imaging system (GE Healthcare Life Sciences, Malborough, MA). Densitometry analysis was performed using ImageJ to quantify protein band intensities.

### Fluorescence Microscopy

Differentiated THP-1 cells grown on glass coverslips were infected with Mtb-GFP for 4 h and then washed with PBS to remove extracellular bacteria. To label lysosomes, differentiated THP-1 cells were pre-loaded with 5 ng/ml Texas-Red-conjugated dextran (10,000 Molecular Weight, Invitrogen), a non-biodegradable polysaccharide that accumulates in the lysosome, 24 h prior to Mtb infection. At 24 h post-infection, cells were fixed with 4% methanol-free formaldehyde, permeabilized using 0.2% TritonX-100, and then blocked with 1% BSA-PBS. Cells were incubated overnight at 4°C in a humidified chamber with a primary polyclonal rabbit antibody to LC3 (MBL International Corporation, Woburn, MA), followed by incubation with Alexa Fluor 568 goat anti-rabbit secondary antibody (Invitrogen). For cells pre-loaded with Texas-Red-conjugated dextran, Alexa Fluor 647 goat anti-rabbit secondary antibody (Invitrogen) was used instead. Nuclei were stained with Hoechst 33342 according to manufacturer’s protocol (NucBlue™ Live ReadyProbes™ Reagent, Invitrogen). Slides were mounted with FluorSave™ reagent (Calbiochem) and imaged using an Axio Imager M2 microscope (Carl Zeiss AG) with 40x objective. Images (z-stacks) were recorded with AxioCam mRm CCD coupled to Zen Blue software and the analyses were performed with Fiji software.

### Statistical Analysis

Data are expressed as the mean ± SEM of three independent biological replicates. Statistical analysis was performed using unpaired *t*-tests (Student’s *t*-test) when comparing two independent cell-lines and paired *t*-tests when comparing within the same cell-line. Values of *p* < 0.05 were considered to be significant. All statistical analyses were performed using GraphPad Prism version 7 (GraphPad Prism Software, San Diego, CA).

## Results

### Intracellular Bacteria Trigger Expression and Activation of PKR in Macrophages

**Figure 1:**
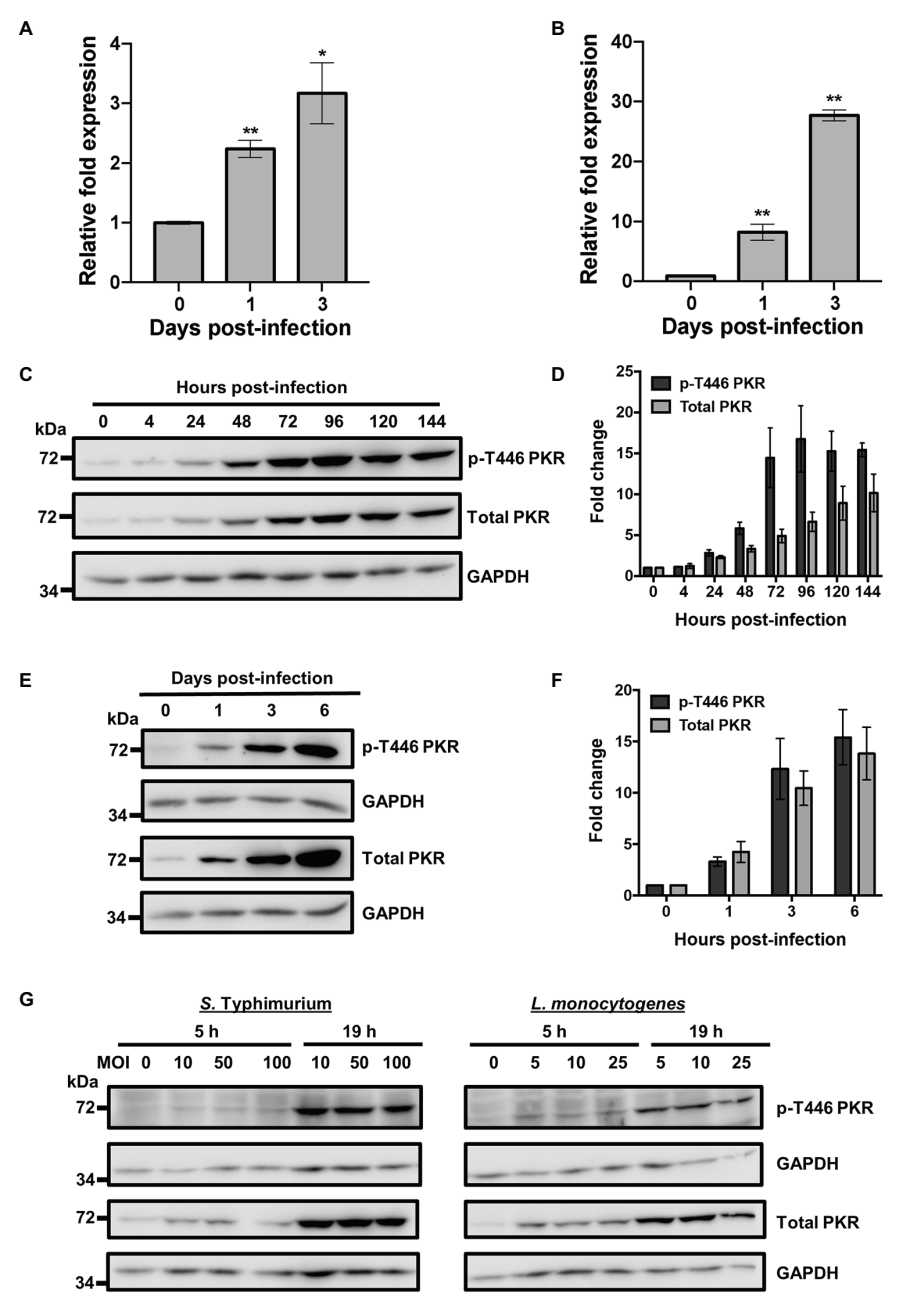
Bacterial infection triggers Protein Kinase R (PKR) expression and activation in macrophages. **(A)** THP-1 macrophages or **(B)** primary human monocyte-derived macrophages (MDMs) were infected with *Mycobacterium tuberculosis* (Mtb) at a multiplicity of infection (MOI) of 5 and quantitative real-time PCR (qRT-PCR) was used to measure the relative expression levels of *EIF2AK2* mRNA at 0, 1, and 3 days post-infection. The ΔΔCT method was used for data analysis by normalizing the Cq values of *EIF2AK2* with two reference genes (*ACTB* and *GAPDH*), and relative fold expression was normalized to uninfected (day 0) macrophages as 1.0. Error bars in **(A,B)** represent the mean ± SEM of three technical replicates. **(C)** THP-1 macrophages were infected with Mtb at an MOI of 5. Cell lysates were prepared at the indicated hours post-infection and total PKR or phosphorylated PKR (p-T446 PKR) protein levels were analyzed by western blotting. The blot shown is representative of three independent experiments. **(D)** Densitometry analysis of the blot in **(C)** was performed by ImageJ to quantify total or p-T446 PKR band intensities as normalized to GAPDH and fold increase of PKR levels is expressed relative to uninfected cells. Error bars represent the mean ± SEM of three independent western blots. **(E)** Primary human MDMs were infected as in **(C)**. Cell lysates were prepared at the indicated days post-infection and total PKR or p-T446 PKR protein levels were analyzed by western blotting. The blot shown is representative of three independent experiments. **(F)** Densitometry analysis of the blot in **(E)** was performed by ImageJ to quantify total or p-T446 PKR band intensities as normalized to GAPDH and fold increase of PKR levels is expressed relative to uninfected cells. Error bars represent the mean ± SEM of three independent western blots. **(G)** THP-1 macrophages were infected with *Salmonella Typhimurium* or *Listeria monocytogenes* at the indicated MOI. Cell lysates were prepared at 5 and 19 h post-infection and total and p-T446 PKR protein levels were analyzed by western blotting. ^*^ *p* < 0.05; ^**^ *p* < 0.01 relative to day 0 controls.

Protein Kinase R has been demonstrated to play a role in the antiviral (Tallóczy et al., 2006) and antiparasitic (Ogolla et al., 2013) response of macrophages. However, knowledge of its role during bacterial infection is surprisingly limited. To investigate whether PKR played a role in the innate immune response to bacterial infections, we measured expression levels of the *EIF2AK2* gene that encodes for PKR following Mtb infection using quantitative real-time PCR (qRT-PCR). Human THP-1 macrophages were infected using the Mtb mc^2^6206 strain, a derivative of Mtb H37Rv (Jain et al., 2014) that has been demonstrated to have similar *in vitro* and intra-macrophage replication rates, similar responses to anti-TB drugs, and induces comparable cytokine production relative to the parent strain (Schaaf et al., 2016; Mouton et al., 2019). By day 3 post-infection, qRT-PCR showed a 3-fold increase in the mRNA expression levels of *EIF2AK2* in THP-1 macrophages (Figure 1A). These findings were then confirmed in primary human MDMs, where mRNA expression of *EIF2AK2* at day 3 post-infection was 28-fold higher compared to uninfected macrophages (Figure 1B). To examine whether the increase in PKR expression translated to the protein level, we measured levels of total PKR protein and phosphorylated PKR protein at threonine 446 (p-T446) following Mtb infection over 6 days. Following activation, PKR homodimerizes, which triggers autophosphorylation on multiple serine and threonine sites, including threonine 446 and 451 (Zhang et al., 2001). These two threonine residues are consistently phosphorylated during PKR activation, thus increasing the catalytic activity of PKR and further stabilizing its homodimerization (Zhang et al., 2001; Dey et al., 2005). Levels of p-T446 were therefore used as a marker for active PKR. Western blot analysis of cell lysates revealed that total and p-T446 levels of PKR start to increase as early as 24 h post-infection (Figure 1C). Total and phosphorylated levels of PKR increased with the length of infection and stabilized at day 4 post-infection, when there was 7-fold and 17-fold higher total and p-T446 PKR expression, respectively, compared to uninfected macrophages (Figure 1D). These findings were confirmed in primary human MDMs (Figure 1E). By day 6 post-infection, levels of total and p-T446 PKR were 14-fold and 15-fold higher, respectively, when compared to uninfected macrophages (Figure 1F). The dramatic increase in PKR expression levels may indicate an innate immune response by macrophages to control bacterial infection. Since PKR expression and activation have not been previously explored in the context of bacterial infections, we sought to further address whether the increase in PKR expression and phosphorylation was a specific response to only Mtb infection. Interestingly, infection by *L. monocytogenes* and *S. Typhimurium* also triggered PKR expression and phosphorylation (Figure 1G), suggesting that regulation of PKR may be a general response to intracellular bacterial infections.

### Genetic Overexpression of PKR Results in Increased and Stable Production of Active PKR During *Mycobacterium tuberculosis* Infection

**Figure 2:**
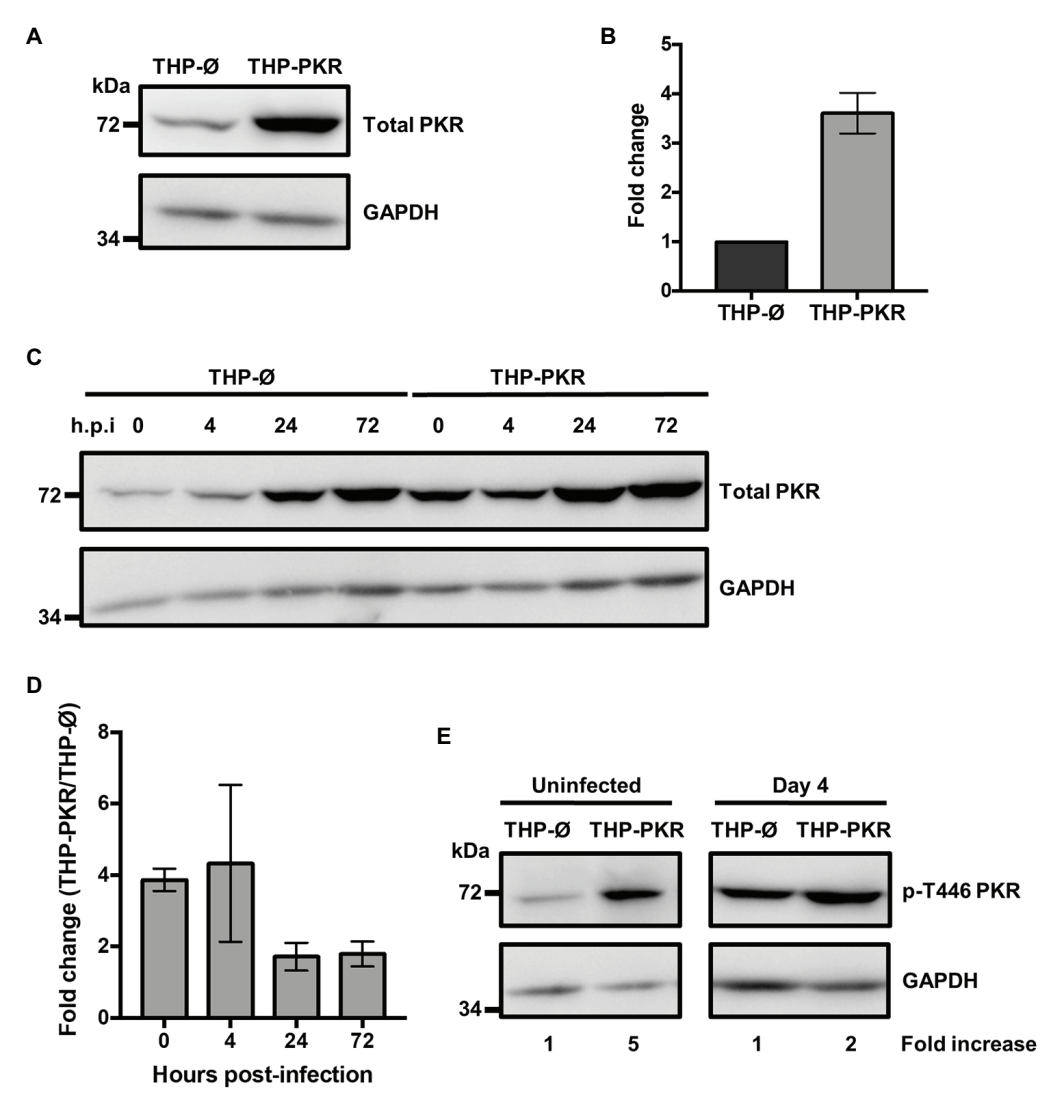
Genetic overexpression of PKR results in increased and stable production of active PKR during *M. tuberculosis* infection. **(A)** PKR protein levels in cell lysates of control THP-1 wild-type (WT) cells transduced with empty vector (THP-Ø) and THP-1 cells transduced with vector overexpressing PKR (THP-PKR) were analyzed by western blotting. **(B)** Densitometry analysis of the blot in **(A)** was performed by ImageJ to quantify PKR band intensities as normalized to GAPDH and fold increase of total PKR levels is expressed relative to THP-Ø cells. **(C)** THP-Ø and THP-PKR macrophages were infected with Mtb at an MOI of 5. Cell lysates were prepared at the indicated hours post-infection (h.p.i) and total PKR protein levels were analyzed by western blotting. **(D)** Densitometry analysis of the blot in **(C)** was performed by ImageJ to quantify PKR band intensities as normalized to GAPDH and fold change of total PKR levels in THP-PKR macrophages relative to THP-Ø macrophages is shown. **(E)** THP-Ø and THP-PKR macrophages were infected as in **(C)**. Cell lysates were prepared at the indicated times post-infection and p-T446 PKR protein levels were analyzed by western blotting. Fold increase of protein expression in THP-PKR macrophages is shown relative to THP-Ø macrophages at the corresponding time-points. The blots in this figure are representative of three independent experiments. Error bars represent the mean ± SEM of three independent western blots.

Given a report that deletion of PKR does not affect control of Mtb infection (Wu et al., 2018), along with our observation that PKR expression and activation is triggered during bacterial infection (Figure 1), we speculated that increased PKR expression and phosphorylation in macrophages would be beneficial to the antibacterial response. To examine the role and function of PKR on the antibacterial functions of macrophages, we genetically overexpressed PKR in THP-1 cells. PKR overexpression cells (THP-PKR) were generated by transducing THP-1 cells with a retroviral expression vector encoding the *EIF2AK2* gene. Western blot analysis confirmed that THP-PKR cells have increased expression of PKR (Figure 2A). Indeed, THP-PKR cells produced 4-fold higher PKR protein levels compared to THP-1 cells transduced with an empty vector (THP-Ø), which were used as control cells throughout this study (Figure 2B). To ensure that overexpression of PKR does not induce any toxic effects in the cells, we compared the proliferation and viability of wild-type (WT), THP-Ø, and THP-PKR macrophages over 72 h, which showed no significant difference between the cell-lines (Supplementary Figure S1). Given that PKR expression is already triggered by Mtb infection in WT THP-1 macrophages (Figures 1C,D), it was difficult to predict whether there would be a discernible difference in PKR expression between Mtb-infected THP-Ø and THP-PKR macrophages. To evaluate this, we infected THP-Ø and THP-PKR macrophages with Mtb and compared the levels of total PKR expression over the course of infection. Western blot analysis revealed that THP-PKR macrophages produced elevated PKR protein levels before and early after infection with Mtb (4-fold increase at 4 h post-infection), compared to THP-Ø macrophages (Figures 2C,D). The increased expression of PKR was also sustained at 24 and 72 h post-infection, when total PKR protein levels remained 2-fold higher in THP-PKR macrophages compared to THP-Ø macrophages (Figures 2C,D). We next questioned whether the increased level of PKR protein observed in THP-PKR macrophages was also phosphorylated. Since PKR expression and activation stabilizes at day 4 post-infection (Figure 1C), we chose this time-point to compare protein levels of p-T446 PKR in THP-Ø and THP-PKR macrophages. Western blot data revealed that at day 4 post-infection, THP-PKR macrophages have 2-fold higher levels of phosphorylated PKR compared to THP-Ø macrophages (Figure 2E). These data collectively show that the generated THP-PKR cells have higher activation and expression levels of PKR in comparison to control cells during basal conditions as well as during Mtb infection. Thus, the generated THP-PKR macrophages provided a suitable cell-line model to examine whether increased expression of PKR functions to improve the cellular antibacterial response against Mtb infection.

### PKR Expression Alters the Immunological Profile of Macrophages During *Mycobacterium tuberculosis* Infection

**Figure 3:**
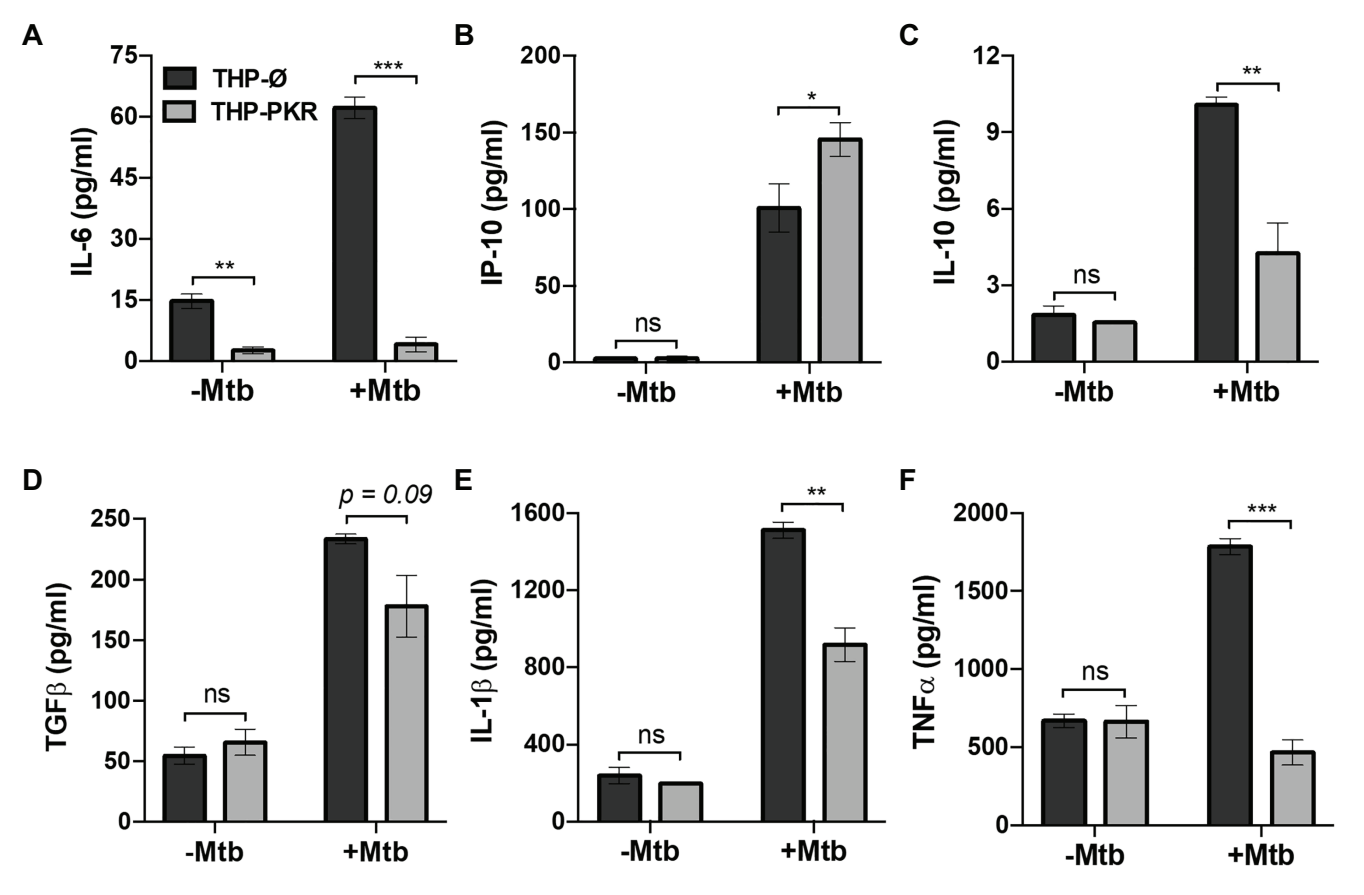
PKR expression alters cytokine production by macrophages during *M. tuberculosis* infection. **(A–F)** THP-Ø and THP-PKR macrophages were infected with Mtb at an MOI of 5 and cell culture supernatant was collected at 24 h post-infection. Production of **(A)** IL-6, **(B)** IP-10, **(C)** IL-10, **(D)** TGFβ, **(E)** IL-1β, and **(F)** TNFα was measured in cell culture supernatant using antibody-based bead multiplex assays. Error bars represent the mean ± SEM of three independent biological replicates. ^*^p < 0.05; ^**^p < 0.01; and ^***^p < 0.001.

To determine whether sustained PKR expression and activation impacts the antibacterial response of macrophages, we first examined the effect of PKR expression on the production of cytokines during Mtb infection. Antibody-based bead multiplex assays were performed to compare cytokine and chemokine production by uninfected and Mtb-infected THP-Ø and THP-PKR macrophages using culture supernatants collected at 24 h post-infection. Consistent with previous reports (Dutta et al., 2012; Sun et al., 2016), Mtb infection induced production of IL-6, IP-10, IL-10, TGFβ, IL-1β, and TNFα by THP-Ø control macrophages (Figures 3A–F). Importantly, overexpression of PKR significantly altered production of cytokines that are of relevance to Mtb infection. The most striking observation was that Mtb-infected THP-PKR macrophages produced 15-fold lower levels of IL-6 compared to THP-Ø macrophages (Figure 3A). Mtb is reported to induce production of IL-6 by infected macrophages to inhibit IFNγ-mediated macrophage activation and autophagy (Nagabhushanam et al., 2003; Dutta et al., 2012). Remarkably, Mtb-infected THP-PKR macrophages produced even less IL-6 than uninfected THP-Ø macrophages (Figure 3A). Mtb-infected THP-PKR macrophages also produced increased levels of IP-10 (Figure 3B), an IFNγ-inducible chemokine. IP-10 functions as a chemoattractant for activated T cells and monocytes and has also been correlated to autophagy induction (Chu et al., 2017). Importantly, a role for IP-10 in restricting Mtb growth has been reported (Palucci et al., 2019). Consistent with this, Mtb-infected THP-PKR macrophages also produced higher levels of IFNγ compared to THP-Ø macrophages (Supplementary Figure S2A). IFNγ is a key cytokine in Mtb immunity due to its critical role in macrophage activation to enhance phagocytosis, apoptosis, autophagy, and the production of reactive nitrogen species (Flynn et al., 1993; Herbst et al., 2011; Dutta et al., 2012). We also observed that THP-PKR macrophages produced decreased levels of IL-10 and TGFβ compared to THP-Ø macrophages (Figures 3C,D). IL-10 and TGFβ are reported to be conducive to Mtb survival due to their inhibitory effect on pro-inflammatory cytokine production and T cell activation (Othieno et al., 1999). Furthermore, IL-10 is reported to inhibit autophagy in Mtb-infected macrophages (Upadhyay et al., 2018). Infected THP-PKR macrophages also produced lower levels of IL-1β and TNFα compared to control macrophages (Figures 3E,F), two cytokines that are critical for host resistance to Mtb due to their pro-inflammatory effects and their role in activating macrophage cell death (Flynn et al., 1995; Keane et al., 1997; Mayer-Barber et al., 2010; Jayaraman et al., 2013). Levels of IL-2, MCP-1, IL-4, IL-8, and IL-17A were also measured but their production did not differ significantly between THP-Ø and THP-PKR macrophages (Supplementary Figures S2B–F). Collectively, our data show that THP-PKR macrophages produced lower levels of cytokines that are permissive for Mtb infection (IL-6, IL-10, and TGFβ) and increased levels of anti-mycobacterial cytokines (IFNγ and IP-10), which supports our hypothesis that sustained PKR expression could enhance the anti-Mtb response in macrophages.

### PKR Is Required to Control *Mycobacterium tuberculosis* Survival in Macrophages

**Figure 4:**
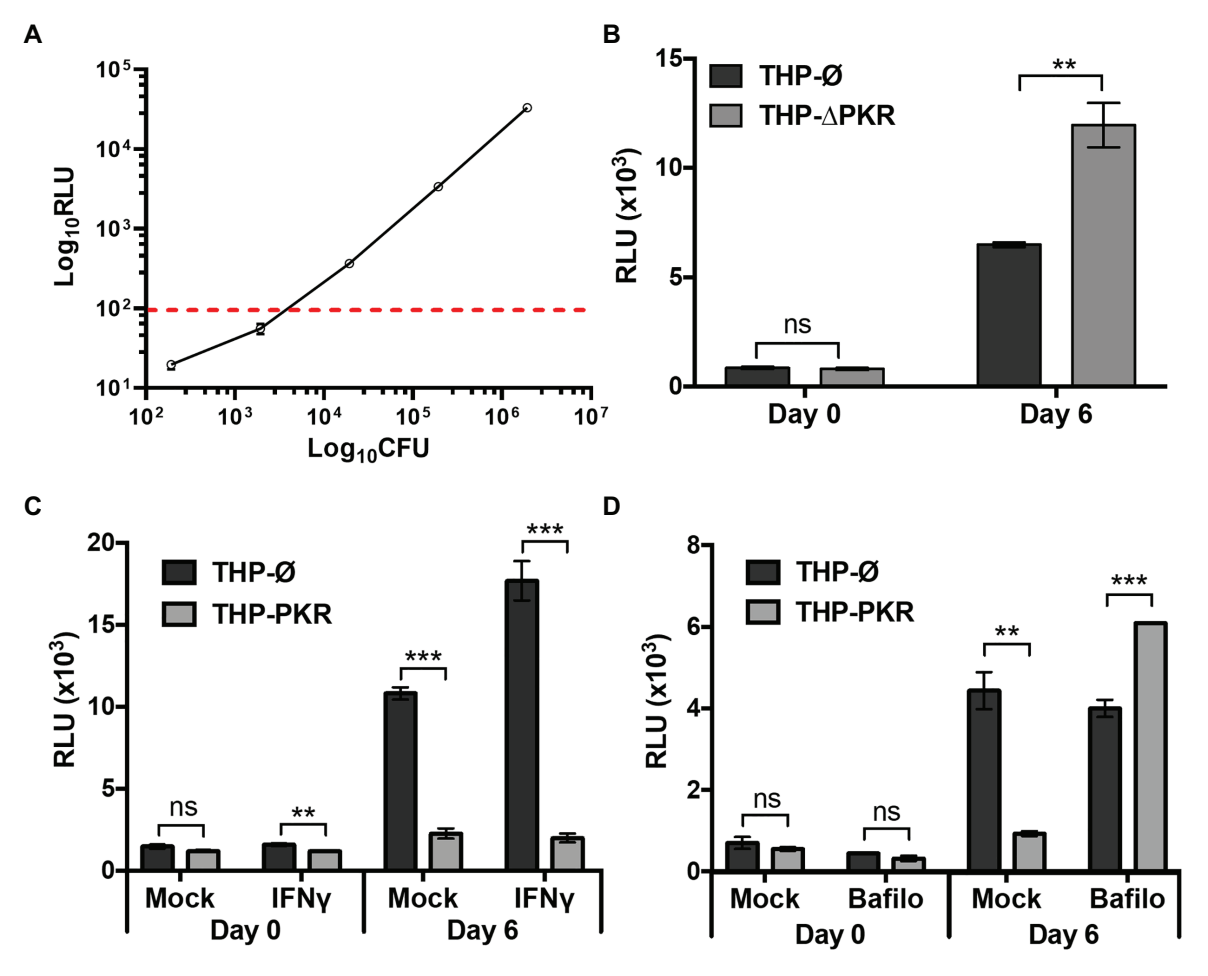
PKR is required to control *M. tuberculosis* survival in macrophages. **(A)** Ten-fold serial dilutions of pre-determined Mtb-luciferase stocks were made in triplicate. The luciferase activity of the Mtb dilutions was measured using a microplate reader to detect luminescence. RLU, relative light units. The Mtb dilutions were also inoculated on agar plates and incubated at 37°C for 3 weeks prior to counting colony forming units (CFU). The dashed line indicates the sensitivity threshold for a linear relationship between CFU and RLU. **(B)** THP-Ø and THP-∆PKR macrophages were infected with Mtb-luciferase at an MOI of 5. Cells were lysed at the indicated time post-infection and the resultant luminescence signal (RLU) was measured. **(C,D)** THP-Ø and THP-PKR macrophages were **(C)** mock-treated or pre-treated for 24 h with IFNγ (100 ng/ml), or **(D)** mock-treated or pre-treated for 1 h with bafilomycin A1 (12 nM) as indicated. Macrophages were infected as in **(B)** and lysed at the indicated time post-infection to measure RLU. IFNγ and bafilomycin A1 were maintained in the medium for the duration of the experiments and replenished every second day. Error bars represent mean ± SEM of three independent biological replicates. ^**^p < 0.01; and ^***^p < 0.001.

Since the cytokine profiling data suggested that PKR expression could enhance the antibacterial response of Mtb-infected macrophages, we next sought to determine whether PKR is required to limit the intracellular survival of Mtb. To measure the viability of intracellular Mtb, we used a well-characterized luciferase reporter system (Sun et al., 2009; Sorrentino et al., 2016). The luciferase reporter system in mycobacteria is strongly correlative with traditional CFU data obtained by plating serial dilutions of bacteria on solid media. Rigorous studies have demonstrated that the luminescence signal from mycobacteria expressing luciferase maintains a linear relationship with CFU over several orders of magnitude (Andreu et al., 2010; Sharma et al., 2014; Ozeki et al., 2015; Early et al., 2019). To demonstrate that these reports are consistent with our specific firefly luciferase reporter system in Mtb, we compared CFU obtained from plating Mtb-luciferase to luminescence [expressed as relative light units (RLU)] produced by the same sample of Mtb-luciferase. This experiment showed a consistent linear relationship between CFU and RLU within the range of ~100–50,000 RLU, which corresponds to approximately 2,500–1 million CFU (Figure 4A). Given that we routinely obtain RLU signals well within this linear correlative range, the luciferase reporter system is a suitable method for measuring the intracellular survival of Mtb.

To address whether PKR is required for control of Mtb survival in macrophages, we deleted PKR in THP-1 cells (THP-∆PKR) using CRISPR/Cas9-mediated genome editing methods. Western blot analysis confirmed that the THP-ΔPKR cells do not express any PKR protein (Supplementary Figure S3), and these cells exhibit normal cell proliferation and viability compared to control cells (Supplementary Figure S1). Importantly, we observed increased intracellular survival of Mtb in THP-∆PKR macrophages compared to control macrophages as indicated by an 84% increase in luminescence signal (Figure 4B). This observation suggests that PKR expression is required to control Mtb survival in human macrophages, which is consistent but slightly different from the report by Wu et al. (2018), which showed that deletion of PKR does not affect Mtb burden in the mouse model. This data is also consistent with our hypothesis that increased production of PKR could lead to an enhanced anti-mycobacterial effect in macrophages.

We then examined whether THP-1 macrophages overexpressing PKR would be more effective at controlling intracellular Mtb survival. Since Mtb-infected THP-PKR macrophages produced higher levels of IFNγ (Supplementary Figure S2A) and IFNγ-stimulated genes (IP-10, Figure 3B), we also wanted to investigate whether PKR functions through an IFNγ-dependent mechanism. We thus performed bacterial survival assays in either mock-treated or IFNγ-treated THP-PKR and THP-Ø macrophages. At day 6 post-infection, the luminescence signal in the mock-treated condition was 79% lower in THP-PKR macrophages compared to THP-Ø macrophages (Figure 4C). In addition, treatment with IFNγ further reduced the intracellular survival of Mtb in THP-PKR macrophages, resulting in a decrease in luminescence of 89% at day 6 post-infection compared to THP-Ø macrophages (Figure 4C). Although IFNγ treatment is reported to reduce Mtb survival in mouse macrophages (Herbst et al., 2011), IFNγ treatment did not reduce Mtb survival in the THP-Ø control macrophages (Figure 4C). This may be explained by the fact that IFNγ reduces Mtb survival via nitric oxide (NO)-dependent apoptosis, but PMA-differentiated THP-1 macrophages are reported to have limited NO production (Daigneault et al., 2010; Herbst et al., 2011). Altogether, these findings suggest that the function of PKR to limit the survival of Mtb in macrophages could be enhanced by addition of IFNγ. This finding is consistent with the cytokine data demonstrating that THP-PKR macrophages produced significantly lower levels of IL-6 and IL-10 compared to THP-Ø macrophages (Figures 3A,C), which antagonize the macrophage activating effects of IFNγ (Gazzinelli et al., 1992; Nagabhushanam et al., 2003; Upadhyay et al., 2018).

Next, we wanted to determine the specific IFNγ-dependent mechanism responsible for the enhanced ability of PKR overexpressing macrophages to limit Mtb survival. Since IL-6 is reported to inhibit IFNγ-induced autophagy in Mtb-infected macrophages (Dutta et al., 2012), we hypothesized that autophagy could be the mechanism responsible for the reduced Mtb survival in THP-PKR macrophages. This hypothesis is also consistent with our observation that Mtb-infected THP-PKR macrophages produced increased levels of IP-10 (Figure 3B), a chemokine linked to induction of autophagy (Chu et al., 2017). Furthermore, although the role of PKR in autophagy during bacterial infection has not yet been studied, PKR is reported to induce autophagy during viral (Tallóczy et al., 2006) and parasitic (Ogolla et al., 2013) infections. To investigate whether autophagy is the mechanism responsible for the reduced Mtb survival in THP-PKR macrophages, we examined the impact of bafilomycin A1 treatment on the bacterial survival in Mtb-infected macrophages. Bafilomycin A1 is a vacuolar H^+^-ATPase inhibitor that blocks the autophagic flux by inhibiting lysosome acidification (Yamamoto et al., 1998). After determining a non-cytotoxic concentration of bafilomycin A1 (Supplementary Figure S4), we measured the intracellular survival of Mtb in mock-treated or bafilomycin A1-treated macrophages. Consistent with our previous bacterial survival assay (Figure 4C), mock-treated THP-PKR macrophages showed decreased intracellular Mtb survival, with a 79% decrease in luminescence signal compared to THP-Ø macrophages (Figure 4D). Importantly, treatment with bafilomycin A1 completely inhibited the ability of THP-PKR macrophages to limit Mtb survival. Indeed, bafilomycin A1-treated THP-PKR macrophages showed a 52% increase in luminescence signal compared to THP-Ø macrophages (Figure 4D) at day 6 post-infection. These data suggest selective autophagy as a potential mechanism responsible for the enhanced ability of THP-PKR macrophages to control intracellular Mtb replication. However, bafilomycin A1 also inhibits acidification of Mtb-containing phagosomes, a molecular pathway distinct from autophagy (Gutierrez et al., 2004). Therefore, we next sought to examine specific markers of autophagy activation to support this hypothesis.

### PKR Expression Activates the Selective Autophagy Pathway in *Mycobacterium tuberculosis*-Infected Macrophages

**Figure 5:**
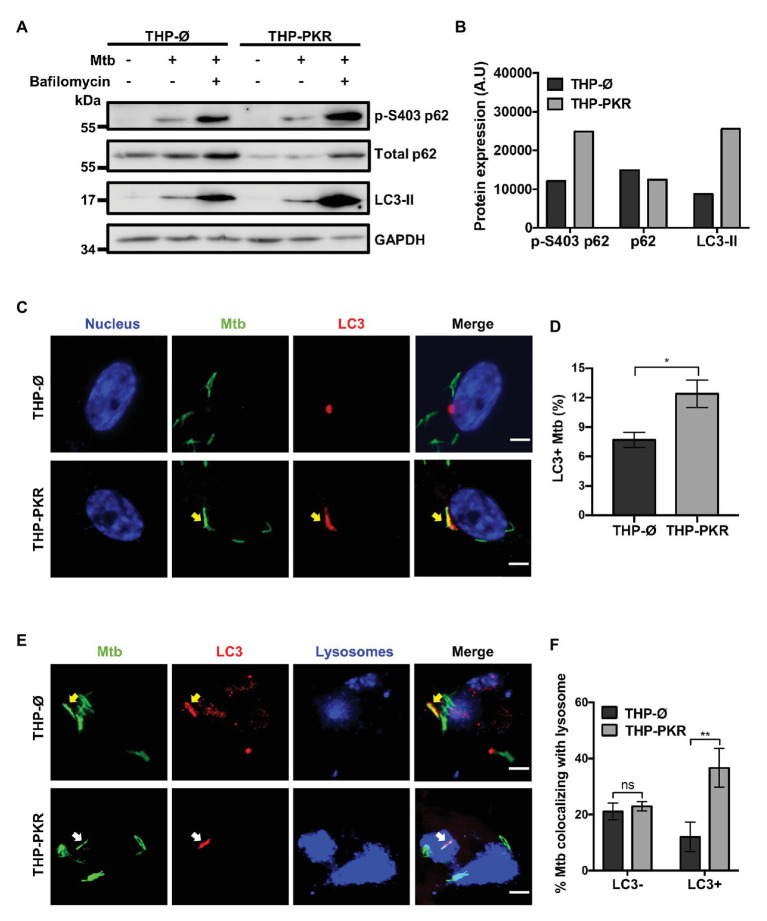
PKR expression activates the autophagy pathway in *M. tuberculosis*-infected macrophages. **(A)** THP-Ø and THP-PKR macrophages were pre-treated with bafilomycin A1 (12 nM) for 1 h where indicated and then infected with Mtb at an MOI of 10. Bafilomycin A1 was maintained in the medium for the duration of the experiment. Cell lysates were prepared at 24 h post-infection and phosphorylated p62 (p-S403 p62), total p62, and LC3-II protein levels were analyzed by western blotting. **(B)** Densitometry analysis of the blot in **(A)** was performed by ImageJ to quantify p-S403 p62, total p62, and LC3-II band intensities in Mtb-infected, bafilomycin A1-treated macrophages as normalized to GAPDH. A.U, arbitrary units. **(C)** THP-Ø and THP-PKR macrophages were infected with Mtb-GFP at an MOI of 10. At 24 h post-infection, cells were fixed, permeabilized, and incubated with an anti-LC3 antibody to visualize autophagosomes. Representative images show nuclei (blue channel), Mtb (green channel), and LC3 (red channel) as detected by fluorescence microscopy. Yellow arrow denotes colocalization between Mtb and LC3. Scale bar, 5 μm. **(D)** Quantification of percent Mtb colocalization with LC3 (LC3^+^Mtb) per total number of intracellular Mtb. A minimum of 20 visual fields, each with 15–30 infected cells, were counted per cell-line. **(E)** THP-Ø and THP-PKR macrophages were loaded with dextran (lysosome marker) for 24 h and then infected and treated as in **(C)**. Representative images show Mtb (green channel), LC3 (red channel), and lysosomes (blue channel) as detected by fluorescence microscopy. Yellow arrow denotes colocalization between Mtb and LC3, and white arrow denotes colocalization of Mtb with both LC3 and lysosomes. Scale bar, 5 μm. **(F)** Quantification of lysosome colocalization with LC3-negative or LC3-positive Mtb is reported as percentage over the total number of LC3-negative or LC3-positive intracellular Mtb, respectively. A minimum of 20 visual fields, each with 15–30 infected cells, were counted per cell-line. Data in **(D,F)** represent the mean ± SEM of all the analyzed visual fields. ^*^p < 0.05; ^**^p < 0.01.

To ascertain that autophagy is indeed the mechanism responsible for reduced Mtb survival in PKR overexpressing macrophages, we examined specific markers of selective autophagy. We compared protein expression levels of p62, phosphorylated p62 (p-S403 p62), and LC3-II in Mtb-infected macrophages treated with bafilomycin A1 (Figure 5A). LC3-II and p62 are well-characterized markers for selective autophagy (Klionsky et al., 2012). LC3-II is extensively used to measure autophagic flux, which is the dynamic process of autophagy that takes into account autophagic degradation activity (Klionsky et al., 2012). It is necessary to use bafilomycin A1 to block the degradation of autophagy markers when performing western blot experiments to enable a true representation of the autophagic flux (Klionsky et al., 2012). An increase in LC3-II expression in bafilomycin A1-treated cells is thus associated with an increase in autophagy. Phosphorylation of serine 403 (p-S403) on p62 regulates selective autophagy through the recognition of ubiquitinated bacteria targeted for autophagic degradation (Matsumoto et al., 2011), and as such, increased levels of p-S403 p62 in bafilomycin A1-treated cells is also an indicator of increased autophagy. Western blot analysis revealed that during Mtb infection, bafilomycin A1-treated THP-PKR macrophages showed 3-fold higher expression levels of LC3-II and 2-fold higher expression of p-S403 p62 compared to THP-Ø macrophages (Figures 5A,B). Therefore, our western blot data suggest that THP-PKR macrophages have increased autophagy activation compared to control macrophages. However, since western blot analysis of cell lysates only measures bulk autophagy and not selective autophagy, we then used fluorescence microscopy to quantify the specific colocalization between intracellular Mtb and LC3. Immunofluorescence microscopy showed that PKR expression induced LC3 association with Mtb (Figure 5C). A significantly higher percentage of LC3^+^ Mtb autophagosomes was observed in THP-PKR macrophages (12%) compared to control THP-Ø macrophages (8%; Figure 5D). We also performed fluorescence microscopy to quantify Mtb and LC3 colocalization in PKR knockout macrophages (Supplementary Figure S5A). A significantly lower percentage of LC3^+^ Mtb autophagosomes was observed in THP-∆PKR macrophages (3%) compared to control cells (6%; Supplementary Figure S5B), further supporting our conclusion that PKR expression is important for selective autophagy and limiting the survival of intracellular Mtb.

Although THP-PKR macrophages had a higher percentage of LC3^+^ Mtb autophagosomes, it was important for us to determine the status of the autophagosomes, since Mtb inhibits autophagolysosome formation by blocking autophagosome fusion with lysosomes (Chandra et al., 2015). THP-Ø and THP-PKR macrophages were pre-loaded with dextran, a lysosome marker (Eissenberg and Goldman, 1988), prior to Mtb infection, and then immunofluorescence microscopy was performed. When specifically counting only Mtb autophagosomes, which are denoted as LC3^+^ Mtb, we found a higher percentage of lysosome colocalization in THP-PKR macrophages (36%) compared to THP-Ø macrophages (12%; Figures 5E,F). This supports our hypothesis that sustained PKR expression enhances the activation of autophagy and promotes increased autophagolysosome fusion. Furthermore, the ability of PKR to increase autophagolysosome fusion explains the reduced bacterial survival observed in THP-PKR macrophages (Figures 4C,D). Importantly, there was no difference in the percentage of lysosome colocalization with LC3-negative Mtb, which are presumed to be located mostly in phagosomes, when comparing THP-PKR and THP-Ø macrophages (Figure 5F). This suggests that the effect of PKR overexpression is specific to the selective autophagy pathway and not the phagosome maturation pathway. Altogether, these findings demonstrate that PKR expression induces selective autophagy in Mtb-infected macrophages, thereby contributing to the reduced intracellular survival of Mtb.

### Overexpression of PKR Does Not Alter Macrophage Cell Death Pathways or Phagocytosis

**Figure 6:**
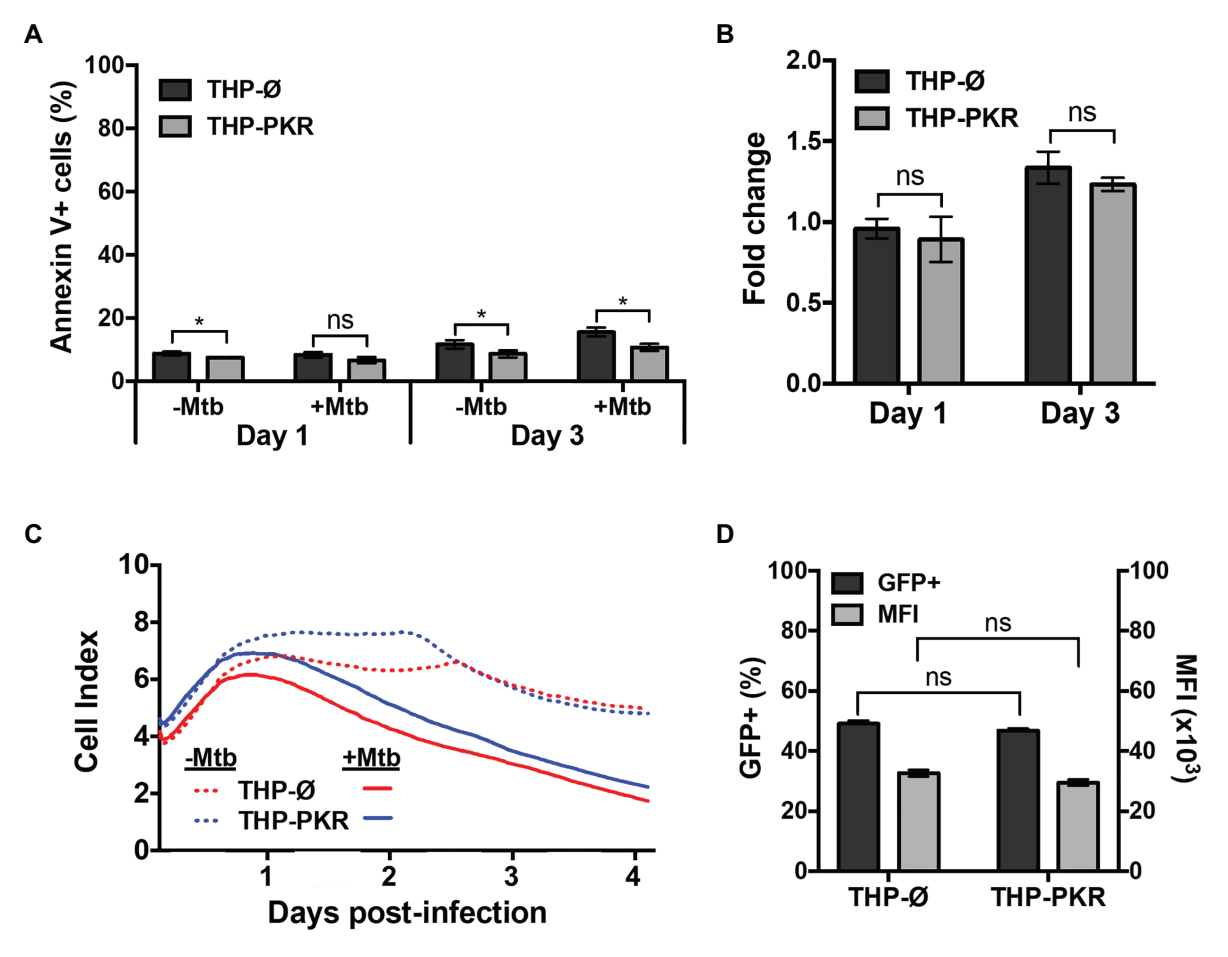
Overexpression of PKR does not affect cell death or phagocytosis. **(A,B)** THP-Ø and THP-PKR macrophages were infected with Mtb at an MOI of 5. At the indicated time post-infection, cells were stained with Annexin V and analyzed by flow cytometry to measure **(A)** the percentage of apoptotic cells, and **(B)** the fold change in apoptosis relative to uninfected cells. **(C)** THP-Ø and THP-PKR macrophages were infected with Mtb at an MOI of 5 and changes in cell adherence were monitored by RTCA as indicated by the Cell Index (CI). CI (viability) was measured continuously for 96 h post-infection. **(D)** THP-Ø and THP-PKR macrophages were infected with Mtb-GFP at an MOI of 10. At 4 h post-infection, PBS washes were performed to remove the extracellular bacteria and the macrophages were analyzed by flow cytometry to quantify the level of phagocytosis using GFP as a marker for internalized Mtb. MFI, mean fluorescent intensity. Error bars indicate mean ± SEM of three independent biological replicates. ^*^p < 0.05.

Our data indicate that sustained expression of PKR induces selective autophagy and reduces intracellular survival of Mtb. However, differences in intracellular bacterial survival between cell-lines can also be caused by changes in cell death or phagocytosis efficiency. Our cytokine profile revealed that THP-PKR macrophages had altered IL-1β and TNFα production compared to control macrophages (Figures 3E,F), cytokines that are positively correlated to macrophage cell death (Keane et al., 1997; Jayaraman et al., 2013). Furthermore, although the role of PKR in macrophage apoptosis during bacterial infection is unclear, it has been well-established to activate stress-induced apoptosis during viral infection or serum starvation (García et al., 2006). As such, we sought to rule-out any potential differences in cell death and phagocytosis between the cell-lines as the cause of the reduced bacterial survival in THP-PKR macrophages to further support our hypothesis that autophagy is the mechanism responsible for this effect. We first examined the impact of PKR expression on the apoptosis of Mtb-infected macrophages. At day 1 post-infection, there was no significant difference in apoptosis levels between THP-Ø and THP-PKR macrophages, as measured by Annexin V assays (Figure 6A). At day 3 post-infection, THP-PKR macrophages showed slightly lower levels of apoptosis (Figure 6A). However, uninfected THP-PKR macrophages also showed a minor decrease in baseline apoptosis compared to THP-Ø macrophages (Figure 6A). When accounting for this, there was no significant difference in fold-change in apoptosis of infected cells relative to uninfected cells when comparing THP-PKR and THP-Ø macrophages (Figure 6B). These results suggest that PKR expression does not induce apoptosis in Mtb-infected macrophages. However, PKR is also reported to induce other forms of cell death, including pyropotosis (Lu et al., 2012) and necroptosis (Thapa et al., 2013). To rule-out all types of cell death caused by modulating PKR, we measured overall cell death using the RTCA assay, a method that quantifies the viability of adherent cells over time (Limame et al., 2012). The RTCA system measures changes in impedance between electrodes at the bottom of E-well plates, which is then translated into a dimensionless value known as the CI. An increase in the CI reflects an increase in macrophage adherence (differentiation), whereas a decrease in the CI reflects the loss of macrophage viability as they detach from the bottom of the well (Schaaf et al., 2017). RTCA data revealed that there was no difference in cell death between Mtb-infected THP-Ø and THP-PKR macrophages over the course of infection (Figure 6C). Therefore, we concluded that overexpression of PKR does not affect cell death in Mtb-infected macrophages.

Lastly, we wanted to ensure that the reduced bacterial survival observed in THP-PKR macrophages was not due to an altered ability to phagocytose Mtb. We infected THP-Ø and THP-PKR macrophages with Mtb expressing GFP for 4 h to allow for bacterial uptake. After 4 h, extensive washes were performed to remove extracellular bacteria and the macrophages were analyzed by flow cytometry to compare the level of phagocytosis using GFP as a marker for internalized Mtb. We observed no significant differences in the percentage of GFP-positive cells or in the numbers of Mtb per macrophage between the two cell-lines (Figure 6D). Therefore, we concluded that the reduced Mtb survival in THP-PKR macrophages is not due to an impact of PKR expression on the phagocytic ability of macrophages. Altogether, these data indicate that the reduced Mtb survival in THP-PKR macrophages is not due to an effect of PKR expression on cell death or phagocytosis, further supporting our hypothesis that autophagy is the predominant mechanism responsible for this effect.

### Pharmacological Activation of Autophagy Enhances the Anti-mycobacterial Effect of PKR Overexpression

**Figure 7:**
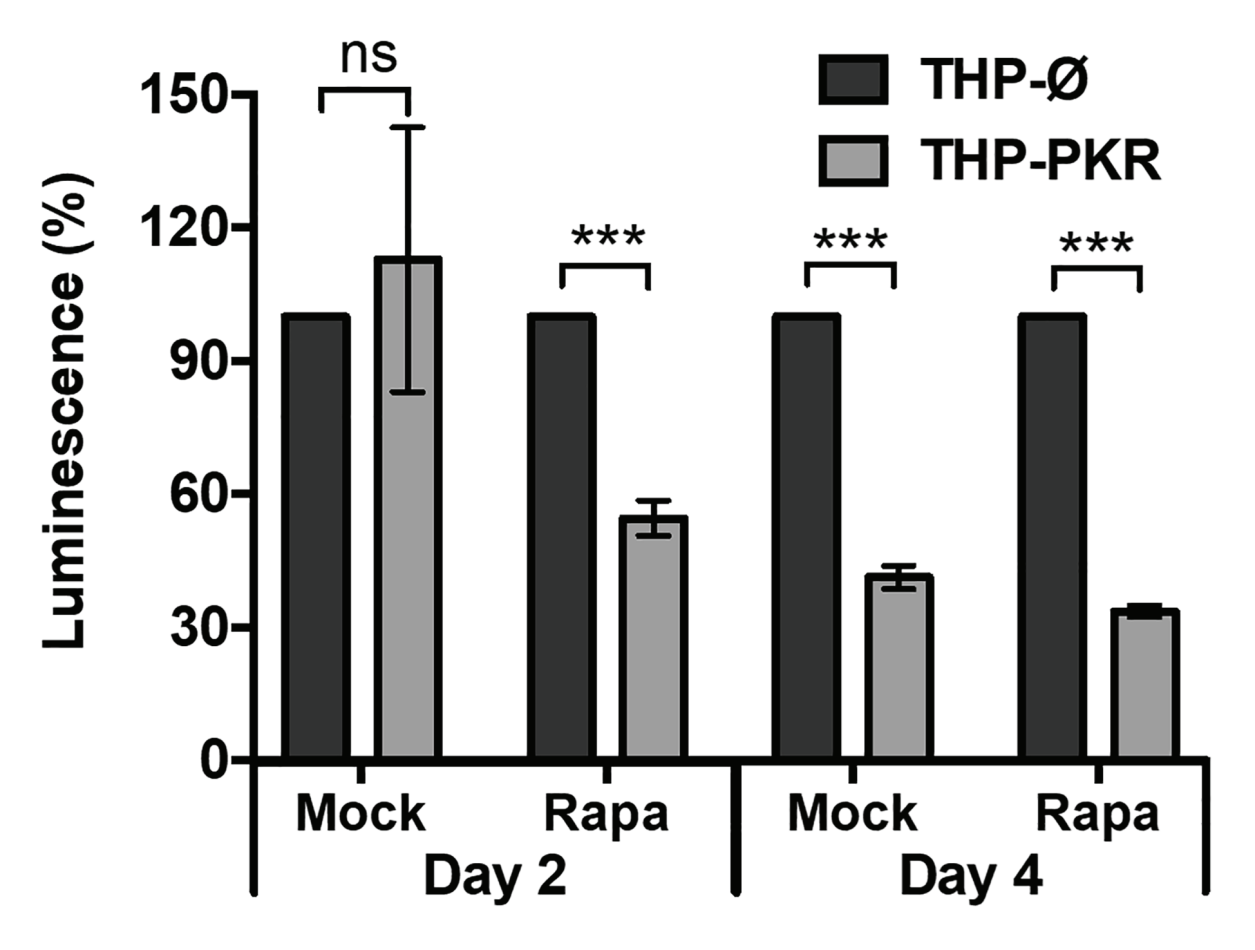
Pharmacological activation of autophagy enhances the anti-mycobacterial effect of PKR overexpression. THP-1 macrophages were mock-treated or pre-treated with rapamycin (50 nM) for 1 h and then infected with Mtb-luciferase at an MOI of 5. Rapamycin was maintained in the medium for the duration of the experiment and replenished every second day. Infected macrophages were lysed at the indicated time post-infection and viable bacteria were determined by measuring the luminescence signal (RLU). RLU signals were normalized to THP-Ø as 100% and reported as % luminescence. Error bars indicate mean ± SEM of three independent biological replicates. ^***^p < 0.001.

Since we observed that genetic upregulation of PKR can limit Mtb survival in macrophages by inducing selective autophagy, we then wanted to determine whether pharmacological activation of autophagy in PKR overexpression macrophages could enhance this effect. Rapamycin, an mTOR inhibitor (Kim and Guan, 2015), was used to pharmacologically activate autophagy. After selecting 50 nM as a non-cytotoxic dose of rapamycin (Supplementary Figure S6), a western blot was performed to verify that this concentration of rapamycin was sufficient to induce LC3-II expression (Supplementary Figure S7). We then measured the intracellular survival of Mtb in mock-treated or rapamycin-treated THP-Ø and THP-PKR macrophages (Figure 7). Interestingly, while mock-treated THP-PKR macrophages did not show a decrease in Mtb survival compared to THP-Ø macrophages at day 2 post-infection, rapamycin-treated THP-PKR macrophages showed a 45% reduction in luminescence relative to similarly treated THP-Ø macrophages (Figure 7). This result showed that rapamycin treatment accelerated and enhanced the effect of PKR overexpression on reducing intracellular Mtb survival. At day 4 post-infection, this effect was less pronounced, as the decrease in luminescence relative to THP-Ø macrophages was more comparable between rapamycin-treated THP-PKR macrophages (66%) and mock-treated THP-PKR macrophages (59%; Figure 7). This suggests that pharmacological activation of autophagy with rapamycin may only limit Mtb survival in the initial phase of infection. Overall, this proof-of-concept experiment supports our hypothesis that PKR functions to decrease Mtb survival through a mechanism dependent on activation of autophagy. Importantly, these findings also suggest that induction of PKR activation/expression in combination with autophagy activation could be a promising strategy for HDT against TB.

## Discussion

There is an urgent need for the development of novel TB therapeutics due to the emergence of antibiotic-resistant Mtb strains. While PKR is established to enhance the macrophage response to viral infection (García et al., 2007), knowledge of its role during bacterial infections is surprisingly limited. Since PKR has been suggested as a potential target for HDT against TB infection (Wu et al., 2012; Tobin, 2015), we sought to investigate the role of PKR in the antibacterial response of Mtb-infected macrophages.

Herein, we provide evidence to suggest that PKR is a promising target for HDT. We show for the first time that PKR expression and activation is triggered by Mtb infection (Figures 1A–F). Our data reveal that PKR enhances the immunological profile of Mtb-infected macrophages by stimulating or inhibiting the production of autophagy-regulating proteins, IP-10, IL-10, and IL-6 (Figures 3A–C). Importantly, we show that PKR expression limits the intracellular survival of Mtb in macrophages through a mechanism dependent on the activation of selective autophagy (Figures 4, 5). A screen of differentially expressed genes in macrophages led Wu et al. (2012) to explore PKR as a potential target for HDT against TB. However, this group investigated the effects of PKR deletion rather than activation, since PKR deletion leaves mice in good health and shows limited or no phenotype upon challenge with certain viruses (Nakayama et al., 2010). While Wu et al. (2012) initially reported that PKR deletion in mice decreases Mtb burden in the lungs, there was a discrepancy between the genetic backgrounds of the mutant and control mice used in the study (Wu et al., 2018). Indeed, their follow-up study using mutant and control mice from the same genetic background revealed that deletion of PKR has no effect on Mtb burden (Mundhra et al., 2018; Wu et al., 2018). This could be due to a redundant role of PKR, since three other EIF2α kinases exist. Indeed, one group showed that EIF2α is still phosphorylated after PKR deletion, albeit to a lesser extent (Zhang and Samuel, 2007). Furthermore, while PKR is reported to induce NF-*κ*B activation (Kumar et al., 1994) – a transcription factor that plays a key role in controlling bacterial load in granulomas during Mtb infection (Fallahi-Sichani et al., 2012) – PKR knockdown did not significantly alter NF-κB activation in response to treatment with TNFα or dsRNA (Zhang and Samuel, 2007). Altogether, these findings suggest that one or more of the other known EIF2α kinases may compensate for the loss of PKR, which could explain why Wu and colleagues did not observe an effect of PKR deletion on Mtb burden. In the human THP-1 macrophage model, we observed that PKR deletion increased the intracellular survival of Mtb (Figure 4B). Based on these results, we speculated that increased PKR expression and activation could instead produce a pro-host effect to reduce intracellular Mtb survival. Since pharmacological activation of PKR has been shown to be well-tolerated in mice (López-Cara et al., 2011), and given that PKR is reported to play a role in the innate immune response to viral and parasitic infection (Tallóczy et al., 2006; Ogolla et al., 2013), we decided it was worthwhile to investigate PKR overexpression as a strategy for TB HDT. Importantly, our findings show that macrophages overexpressing PKR have significantly lower Mtb burden compared to control cells, suggesting that PKR is indeed a promising target for novel TB therapeutics (Figures 4C,D).

Our data showing an antibacterial effect for PKR is consistent with a previous report that PKR is required for the production of anti-mycobacterial cytokines in response to Bacillus Calmette-Guérin (BCG) infection (Cheung et al., 2005), a live attenuated mycobacterium used for tuberculosis vaccination. While Cheung et al. (2005) observed that PKR inhibition by a pharmacological compound or by the transfection of a transdominant negative PKR mutant decreases production of IL-6, IL-10, and TNFα during BCG infection, we report that PKR overexpression decreases the production of these cytokines during Mtb infection. This discrepancy in findings is likely due to the strain of mycobacteria used, since Mtb is capable of escaping to the macrophage cytosol to trigger the cytosolic surveillance and the induction of autophagy (Watson et al., 2012), whereas BCG is known to be incapable of escaping the phagosome (Simeone et al., 2012). In addition, Cheung and colleagues used macrophage precursors, human CD14^+^ blood monocytes, and promonocytic U937 cells, whereas we used primary human monocyte-derived macrophages and differentiated THP-1 macrophages. Cheung et al. (2005) suggest that the effect of PKR on anti-BCG cytokine production is due to downstream activation of NF-κB, since pharmacological inhibition of NF-κB lowered the production of IL-6, IL-,10, and TNFα in response to BCG infection, and treatment with 2-aminopurine (2-AP), a pharmacological inhibitor of PKR, prevented NF-κB activation. However, it is noteworthy that 2-AP has been shown to inhibit other kinases (Posti et al., 1999). Importantly, another group reported that genetic knockdown of PKR did not cause a difference in NF-κB activation compared to control cells (Zhang and Samuel, 2007). This suggests that the effect of 2-AP treatment on NF-κB inhibition observed by Cheung and colleagues may be due to PKR-independent effects of the compound. Further investigation is required to identify the mechanisms responsible for the impact of PKR on anti-mycobacterial cytokine production. Our observation that Mtb-infected macrophages overexpressing PKR have reduced IL-1β (Figure 3E) and TNFα (Figure 3F) production was also unexpected, since these cytokines are reported to assist in Mtb clearance (Flynn et al., 1995; Keane et al., 1997; Mayer-Barber et al., 2010; Jayaraman et al., 2013), yet PKR overexpressing macrophages showed reduced bacterial survival despite lower production of these cytokines. However, Mtb-infected PKR overexpressing macrophages also produced lower amounts of TGFβ and IL-10, two anti-inflammatory cytokines that function to counteract the effects of TNFα and IL-1β (Othieno et al., 1999). As such, it is possible that PKR overexpressing macrophages require a lower threshold of TNFα and IL-1β to prevent hyper-inflammation.

Our data suggest a new role for PKR in regulation of selective autophagy in response to intracellular bacterial infection. Western blot analysis of autophagy markers and immunofluorescence microscopy analysis of LC3 and lysosome colocalization with Mtb revealed that PKR expression induces selective autophagy of Mtb (Figures 5A–F). Inhibition of autophagy also reversed the effects of PKR overexpression on intracellular Mtb survival (Figure 4D). These findings are consistent with previous reports that PKR induces LC3-associated autophagy of *Toxoplasm gondii* (Ogolla et al., 2013) and is required for autophagic degradation of HSV-1 (Tallóczy et al., 2006). Given the key role that IFNγ plays in induction of macrophage autophagy (Gutierrez et al., 2004), and considering that IL-6 inhibits IFNγ-induced autophagy (Dutta et al., 2012), we speculate that the increased activation of autophagy observed in PKR overexpressing macrophages is due, at least in part, to decreased production of IL-6 and increased production of IP-10 and IFNγ by these cells. Consistent with this, we observed that addition of IFNγ enhanced the ability of THP-PKR macrophages to limit the intracellular survival of Mtb (Figure 4C). IFNγ activates autophagy by inducing phosphorylation of mitogen activated protein kinases (MAPK), whereas IL-6 inhibits MAPK phosphorylation to block IFNγ-induced autophagy (Dutta et al., 2012). Interestingly, PKR has been shown to be important for MAPK activation during BCG infection (Cheung et al., 2005) and is also reported to activate MAPK in response to viral infection (Zhang et al., 2009) and stress stimuli (Goh, 2000). As such, it is possible that the induction of autophagy in PKR overexpressing macrophages is dependent on a mechanism involving MAPK activation, whether by a direct effect of PKR on MAPK activation and/or an indirect effect from increased IFNγ production and decreased IL-6 production by PKR overexpressing macrophages.

Collectively, our findings suggest that PKR is a promising target for HDT against TB infection. Since autophagy induction is frequently proposed as an HDT strategy for TB and PKR was observed to induce selective autophagy, we examined whether pharmacological activation of autophagy in combination with PKR overexpression/activation would enhance the antibacterial effect of PKR. Importantly, we observed that rapamycin treatment accelerated and enhanced the decrease in intracellular Mtb survival observed in PKR overexpressing macrophages relative to control cells (Figure 7). This suggests that combination therapy with pharmacological activators of both autophagy and PKR could be a successful strategy for HDT against TB. Although DHBDC is a pharmacological activator of PKR currently available for research use, this compound is not specific since it also activates Protein Kinase R Like Protein (PERK; Bai et al., 2013). Therefore, a search for more specific pharmacological activators of PKR would be desirable. The efficacy of such PKR activators should also be assessed in combination with autophagy activators on Mtb survival both *in vitro* and in mouse models. Furthermore, it will be interesting to ascertain whether the findings in this study extend to other pathogenic intracellular bacteria given that PKR was also highly upregulated in *S. Typhimurium*-infected and *L. monocytogenes*-infected macrophages (Figure 1G).

## Data Availability Statement

The original contributions presented in the study are included in the article/Supplementary Material, further inquiries can be directed to the corresponding author.

## Ethics Statement

The studies involving human participants were reviewed and approved by Ottawa Health Science Network Research Ethics Board. The patients/participants provided their written informed consent to participate in this study.

## Author Contributions

RS and JS conceived and designed experiments, analyzed data, and wrote and edited the manuscript. RS, SB, NR, and TC performed experiments. All authors contributed to the article and approved the submitted version.

### Conflict of Interest

The authors declare that the research was conducted in the absence of any commercial or financial relationships that could be construed as a potential conflict of interest.

## Footnotes

^1^http://n2t.net/addgene:52961

^2^http://n2t.net/addgene:12260
