# Supplementary material for: Protein Kinase R Restricts the Intracellular Survival of Mycobacterium tuberculosis by Promoting Selective Autophagy
Source: Front Microbiol. 2021 Jan 22;11:613963. doi: 10.3389/fmicb.2020.613963 (PMC7862720; doi:10.3389/fmicb.2020.613963)
Supplement: Supplementary file 1 [file Data_Sheet_1.pdf]

Supplementary Material

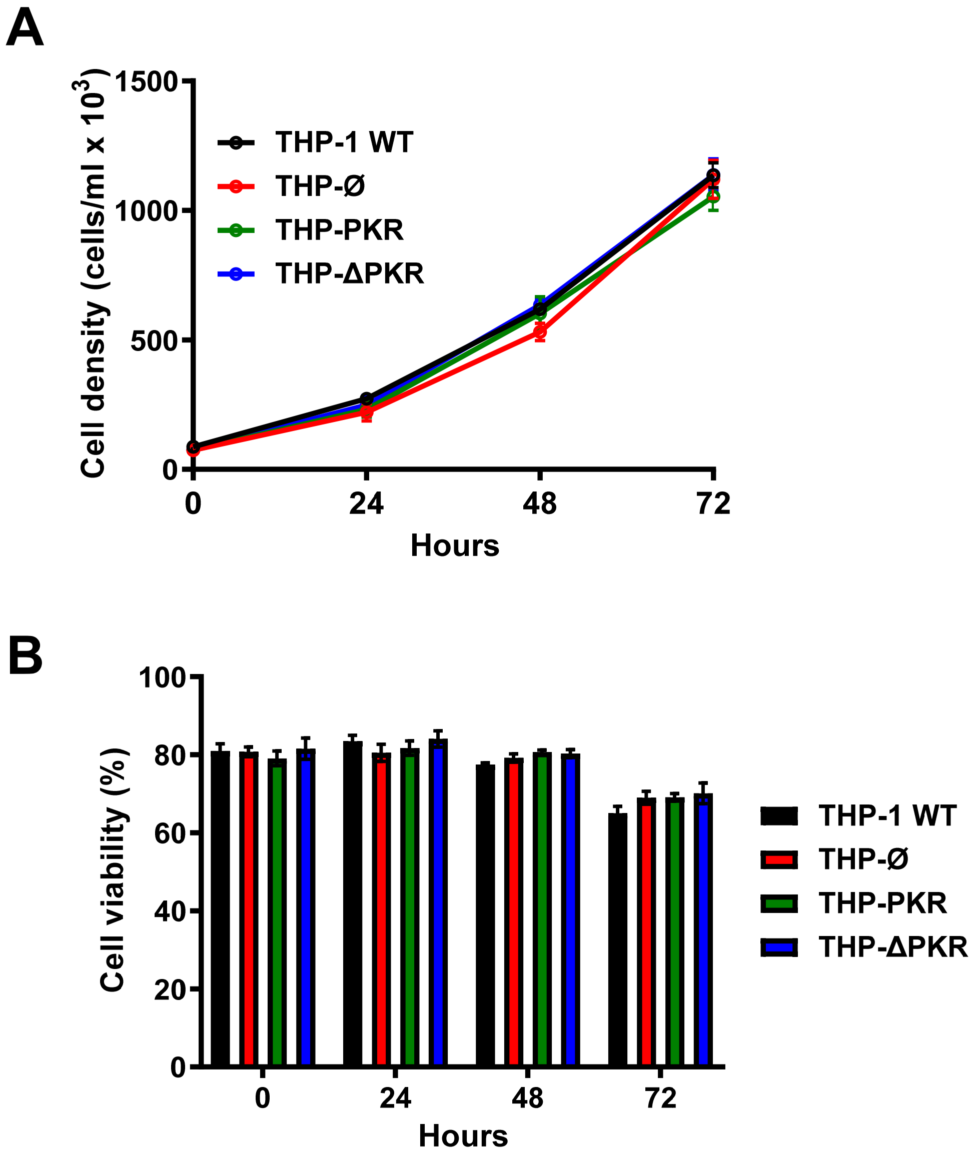

Supplementary Figure 1. Genetic modulation of PKR expression does not impact proliferation or viability of THP-1 cells. THP-1 WT, THP-Ø, THP-PKR, and THP-ΔPKR cells were synchronized to a density of 100,000 live cells per mL at time zero and allowed to grow in culture for a period of 72 hours. Every 24 hours, flow cytometry was performed to measure **(A)** cell density and **(B)** cell viability, as determined by scattering properties. Error bars represent the mean ± SEM of three biological replicates.

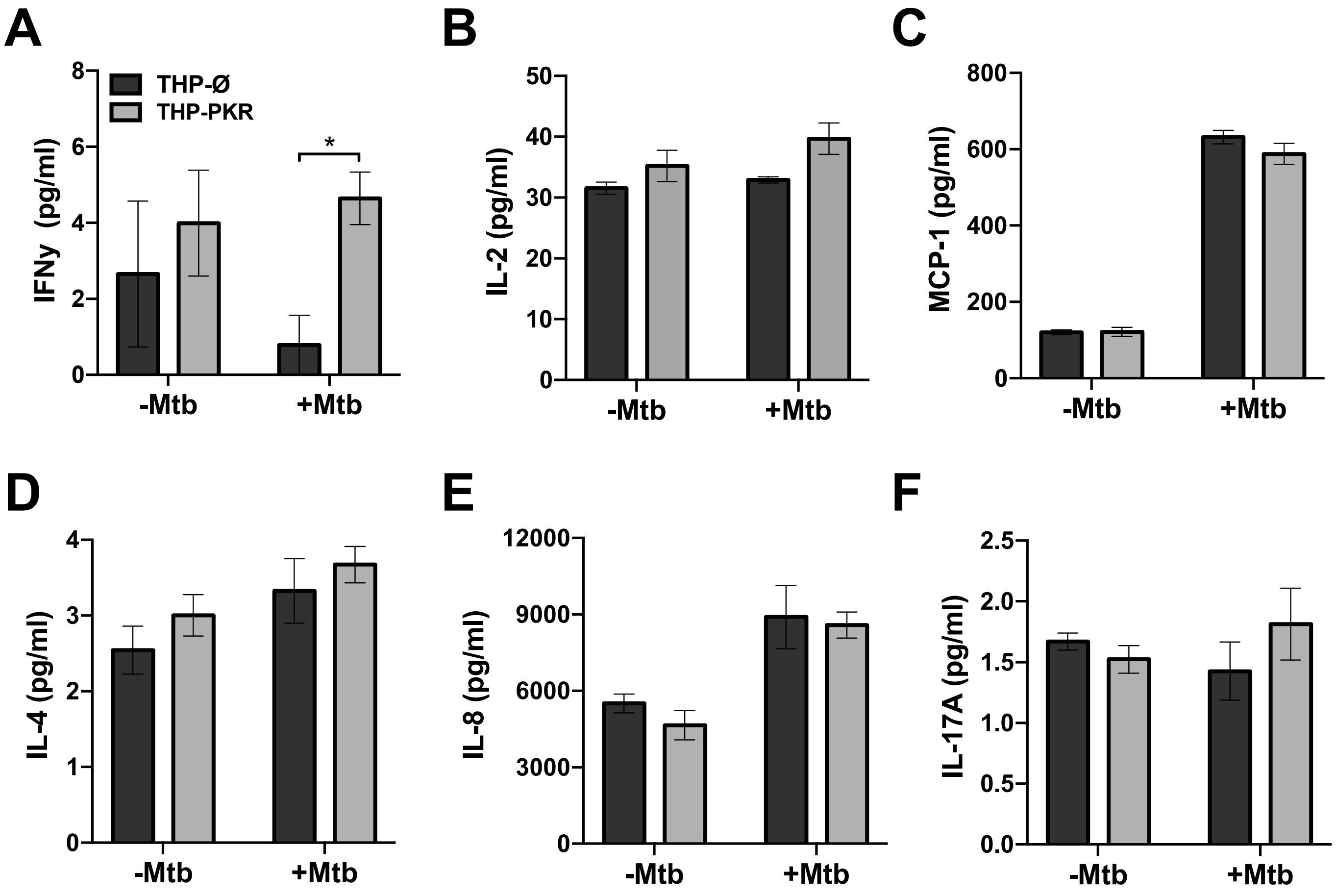

Supplementary Figure 2. Cytokine production in macrophages during *M. tuberculosis* infection. (A-F) THP-Ø and THP-PKR macrophages were infected with Mtb at an MOI of 5 and cell culture supernatant was collected at 24 h post-infection. Production of **(A)** IFNγ **(B)** IL-2, **(C)** MCP-1, **(D)** IL-4, **(E)** IL-8, and **(F)** IL-17A was measured in cell culture supernatant using antibody-based bead multiplex assays. Error bars represent the mean ± SEM of three independent biological replicates. $^*p < 0.05$.

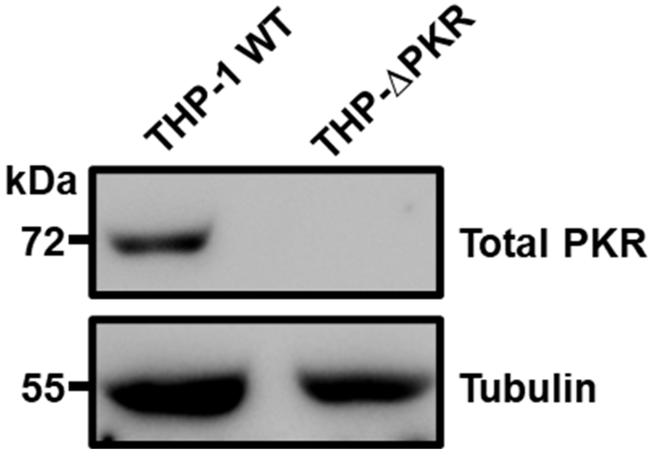

Supplementary Figure 3. Generation of THP-ΔPKR cells. THP-1 cells were transduced with a lentiviral vector encoding a single guide RNA targeting exon 2 of the *EIF2AK2* gene to generate PKR knockout (THP-ΔPKR) cells. Cell lysates were prepared and total PKR protein levels were analyzed by western blotting. The western blot shown is representative of 3 independent experiments.

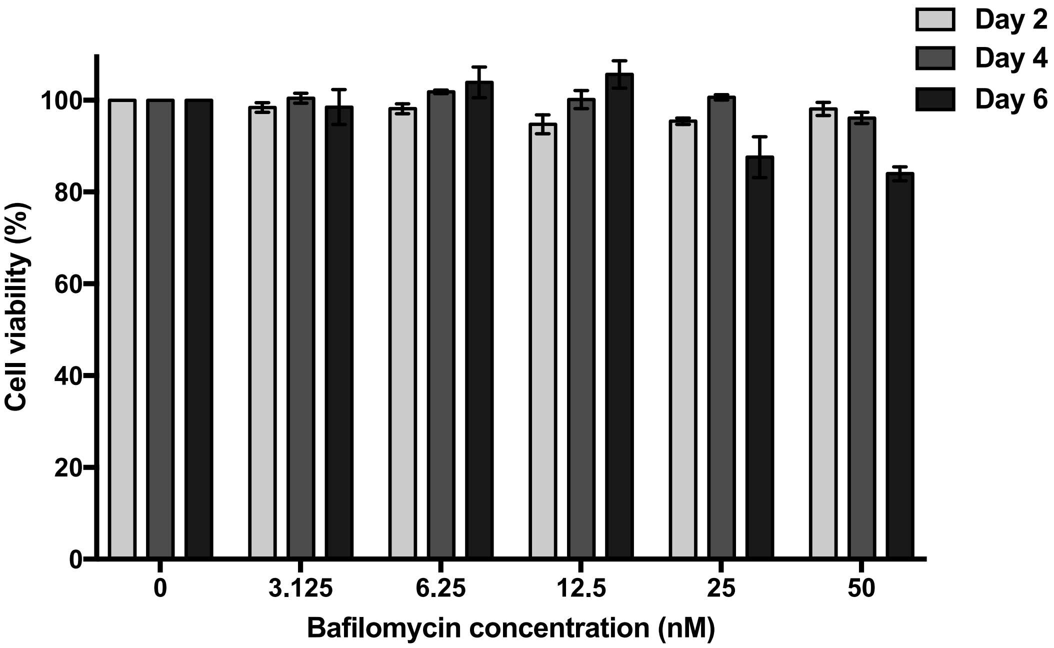

Supplementary Figure 4. Cytotoxicity of bafilomycin A1. THP-Ø macrophages were treated with bafilomycin A1 at the indicated concentrations. At the indicated time-points, resazurin was added to the wells and fluorescence was measured after a 4 h incubation. Fluorescence values were normalized to untreated cells as 100% and reported as % cell viability. Error bars represent the mean ± SEM of three biological replicates.

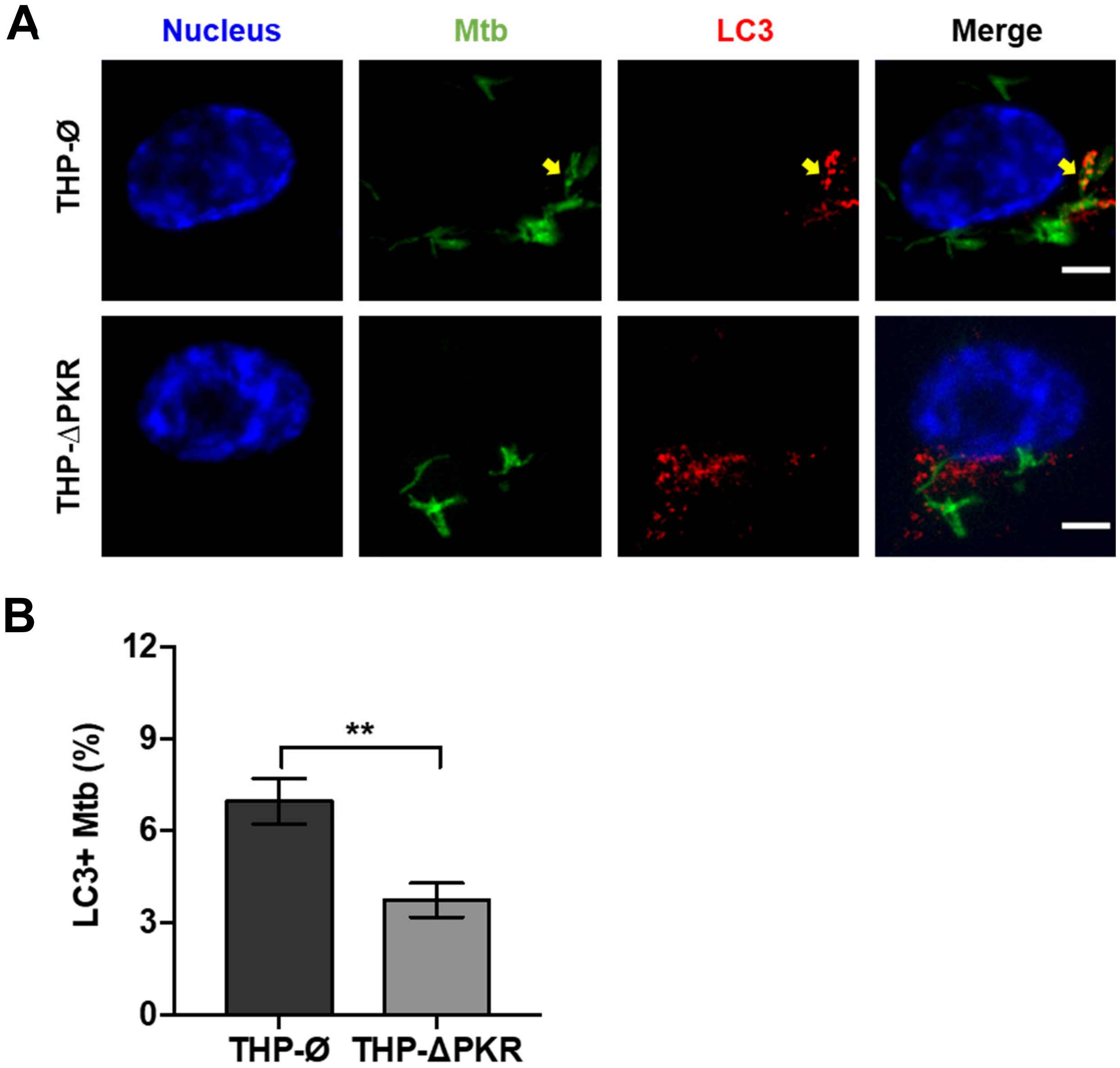

Supplementary Figure 5. Deletion of PKR inhibits selective autophagy of Mtb. (A) THP-Ø and THP-ΔPKR macrophages were infected with Mtb-GFP at an MOI of 10. At 24 h post-infection, cells were fixed, permeabilized, and incubated with an anti-LC3 antibody to visualize autophagosomes. Representative images show nuclei (blue channel), Mtb (green channel), and LC3 (red channel) as detected by fluorescence microscopy. Yellow arrow denotes colocalization between Mtb and LC3. Scale bar, 5 µm. **(B)** Quantification of percent Mtb colocalization with LC3 ($LC3^{+}$Mtb) per total number of intracellular Mtb. A minimum of 20 visual fields, each with 15-30 infected cells, were counted per cell-line. Error bars represent the mean ± SEM of all the analyzed visual fields. $^{**}p < 0.01$.

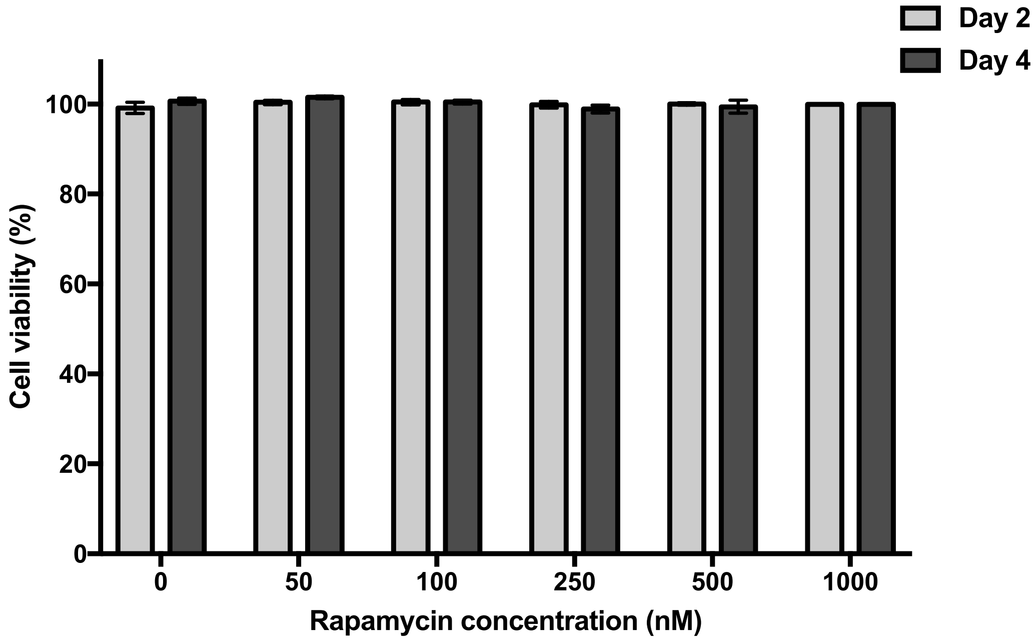

Supplementary Figure 6. Cytotoxicity of rapamycin. THP-Ø macrophages were treated with rapamycin at the indicated concentrations. At the indicated time-points, resazurin was added to the wells and fluorescence was measured after a 4 h incubation. Fluorescence values were normalized to untreated cells as 100% and reported as % cell viability. Error bars represent the mean ± SEM of three biological replicates.

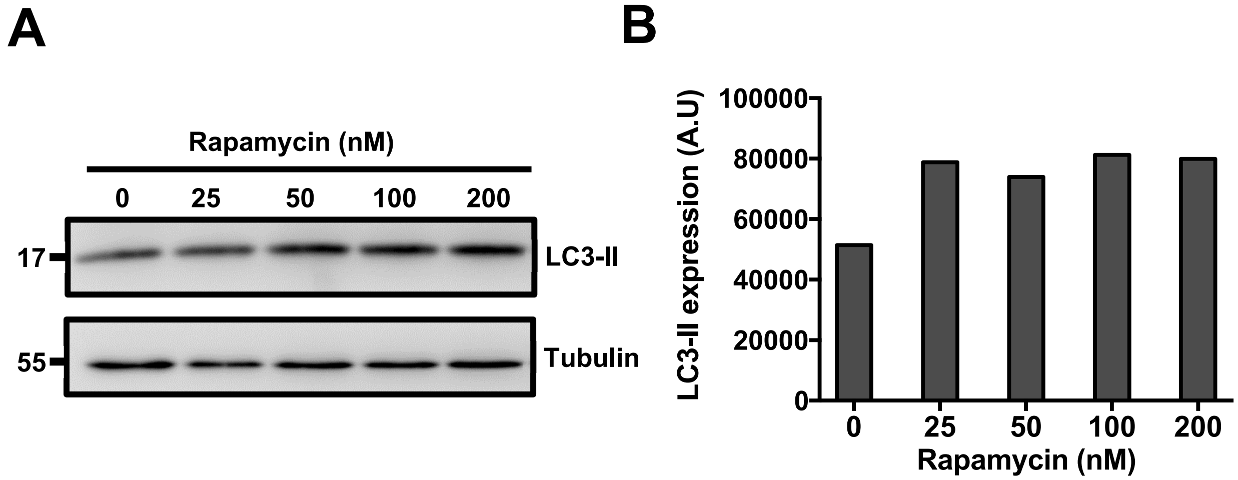

Supplementary Figure 7. Induction of LC3-II expression by rapamycin treatment. (A) THP-1 macrophages were treated with rapamycin at the indicated concentrations. Cell lysates were prepared at 24 h post-treatment and LC3-II protein levels were analyzed by western blotting. **(B)** Densitometry analysis of the blot in (A) was performed by ImageJ to quantify LC3-II band intensities in rapamycin-treated macrophages as normalized to tubulin. A.U., arbitrary units.
